# Outcomes of early versus late radiotherapy in grade 2 meningiomas: a National retrospective analysis from the TROD neuro-oncology group

**DOI:** 10.1007/s11060-026-05590-8

**Published:** 2026-05-05

**Authors:** Volkan Demircan, Ertuğrul Şentürk, Evrim Tezcanlı, Petek Erpolat, Züleyha Akgün, Serra Kamer, Necla Gürdal, Beyhan Ceylaner Bıçakçı, Burak Erdemci, Nuri Kaydıhan, Banu Atalar

**Affiliations:** 1 Bahçesehir University Radiation Oncology Department, Istanbul, Turkey; 2 Gazi University Radiation Oncology Department, Ankara, Turkey; 3 Acıbadem University Altunizade Hospital Radiation Oncology Department, Istanbul, Turkey; 4 Memorial Sisli Hospital Radiation Oncology Department, Istanbul, Turkey; 5 Ege University Radiation Oncology Department, Izmir, Turkey; 6 Cemil Taşçıoğlu City Hospital Radiation Oncology Department, Istanbul, Turkey; 7 Kartal Dr. Lütfi Kırdar City Hospital Radiation Oncology Department, Istanbul, Turkey; 8 Ataturk University Radiation Oncology Department, Erzurum, Turkey; 9 Istanbul Arel University Memorial Bahcelievler Hospital Radiation Oncology Department, Istanbul, Turkey; 10 Acıbadem Maslak Hospital, Istanbul, Turkey; 11 Present Address: Anadolu Medical Center Hospital Radiation Oncology Department, Istanbul, Turkey

**Keywords:** Grade 2 meningioma, Radiotherapy, Surgery, SRS, RT timing

## Abstract

**Purpose:**

The optimal timing of radiotherapy (RT) in patients with WHO grade 2 meningiomas remains controversial, particularly following gross total resection (GTR). This multicenter study compared long-term outcomes of early RT versus late RT, focusing on progression-free survival (PFS), overall survival (OS), and tumor-related mortality.

**Methods and materials:**

This retrospective multicenter cohort study included 263 adult patients with histopathologically confirmed WHO grade 2 meningiomas treated between 2005 and 2023. Patients were stratified by RT timing: early adjuvant RT after initial surgery versus late RT administered after radiologic or clinical progression. Outcomes were analyzed in the overall cohort and in a prespecified GTR subgroup. PFS and OS were estimated using the Kaplan–Meier method. Tumor-related mortality was assessed using cause-specific Cox and Fine–Gray competing-risk models.

**Results:**

With a median follow-up exceeding 7 years, early RT was associated with a significant improvement in PFS compared with late RT. In the overall cohort, 10-year PFS was 84.8% with early RT versus 18.6% with late RT (*p* < 0.001). Among patients undergoing GTR, 10-year PFS remained high with early RT (89.3%) but declined markedly after late RT (18.8%; *p* < 0.001). RT timing was the strongest independent predictor of PFS in multivariable models, although its effect attenuated over time. Tumor-related mortality was approximately sixfold higher in the late RT group (16.7% vs 2.7%; *p* = 0.011), whereas all-cause mortality did not differ significantly (24.2% vs 14.7%; *p* = 0.198). Mitotic count and Ki-67 index were independently associated with early and long-term PFS, whereas male sex, older age, and subtotal resection predicted tumor-related mortality.

**Conclusions:**

Early RT provides a durable and clinically meaningful PFS benefit over late RT in WHO grade 2 meningiomas, including after GTR, without conferring an OS advantage. These findings emphasize the importance of RT timing and tumor biology in postoperative risk stratification and support consideration of early RT in selected patients, pending results from randomized trials.

**Supplementary Information:**

The online version contains supplementary material available at 10.1007/s11060-026-05590-8.

## Key points

Early RT significantly improved progression-free survival in grade 2 meningiomas.

10-year PFS: 84.8% with early RT vs 18.6% with post-recurrence RT.

PFS benefit of early RT persisted after gross total resection.

Ki-67 index and mitotic count predicted progression risk.

Early RT improved disease control but not overall survival.

**Supplementary Information:**

The online version contains supplementary material available at 10.1007/s11060-026-05590-8.

## Importance of Study

The optimal timing of radiotherapy (RT) following surgery for WHO grade 2 meningiomas remains one of the most debated issues in neuro-oncology, particularly after gross total resection. In the absence of definitive randomized evidence, clinical practice varies widely between institutions, with some centers favoring immediate adjuvant RT and others reserving RT for recurrence. In this large national multicenter cohort, early RT was associated with a substantial and durable improvement in long-term progression-free survival compared with delayed RT administered at recurrence. Notably, this benefit persisted even among patients who underwent gross total resection, a population in which the role of adjuvant RT is particularly controversial. These findings provide important real-world evidence supporting the consideration of early postoperative RT in selected patients with grade 2 meningioma while awaiting the results of ongoing randomized trials.

**Supplementary Information:**

The online version contains supplementary material available at 10.1007/s11060-026-05590-8.

## Introduction

Meningiomas are classified into three histopathologic grades: grade 1 (benign), grade 2 (atypical), and grade 3 (anaplastic). In the WHO 2000 classification, grade 2 meningiomas comprised approximately 4–5% of all meningiomas [1]. Subsequent revisions led to a marked increase in reported incidence, reaching 22% in the 2007 classification. In the WHO 2016 classification, despite the incorporation of brain invasion as a diagnostic criterion for grade 2 disease, the incidence remained relatively stable at approximately 25% [2]. Most recently, the WHO 2021 classification integrated molecular markers, including TERT promoter mutations, CDKN2A/B homozygous deletion, H3K27me3 loss, and DNA methylation profiling, enabling more refined risk stratification [3–7].

While management strategies for grade 1 and grade 3 meningiomas are well defined, the optimal treatment approach for grade 2 meningiomas remains controversial, particularly with respect to postoperative radiotherapy (RT). Current EANO (European Association of Neuro-Oncology) and NCCN (National Comphrenesive Cancer Network) guidelines recommend surgery or observation for grade 1 meningiomas and adjuvant RT for grade 3 disease. For grade 2 meningiomas, adjuvant RT is generally recommended following subtotal resection in patients with good performance status. However, the role of adjuvant RT after gross total resection (GTR) remains uncertain [8, 9].

Historical data suggest that 3-year progression-free survival (PFS) rates are approximately 70% with GTR alone and increase to nearly 90% with the addition of adjuvant RT [10]. Prospective phase II data further support this observation. In RTOG (Radiation Therapy Oncology Group) 0539, patients with intermediate-risk disease (grade 2 meningiomas after GTR or recurrent grade 1 meningiomas) achieved a 3-year PFS of 93.8% with adjuvant RT, whereas patients with high-risk disease (grade 2 meningiomas after subtotal resection) had a substantially lower 3-year PFS of 58.8%, despite dose escalation (60 Gy vs 54 Gy) [11, 12]. Similarly, the EORTC (European Organisation for Research and Treatment of Cancer) 22,042– 26,042 trial reported a 3-year PFS of 88.7% with adjuvant RT to 60 Gy in patients with grade 2 meningiomas following GTR [13]. Notably, both trials were single-arm phase II studies, and level I evidence from randomized trials is currently lacking.

The ROAM/EORTC 1308 [14] trial has completed accrual, and results are awaited. In parallel, the NRG BN003 [15] randomized trial is evaluating the benefit of adjuvant RT following GTR in grade 2 meningiomas.

While definitive randomized data are still forthcoming, there is currently no consensus regarding the use or optimal timing of adjuvant RT following GTR in grade 2 meningiomas, resulting in substantial variability in institutional practice and multidisciplinary decision-making. Therefore, the present study aims to report national outcomes for patients with grade 2 meningiomas and to compare long-term disease control according to the timing of RT.

## Methods

### Study design and setting

This multicenter retrospective cohort study was designed to evaluate the impact of RT timing on long-term oncologic outcomes in patients diagnosed with grade 2 meningiomas. The dataset was retrospectively collected from institutional databases of radiation oncology centers across Turkey. The study period spanned from 2005 to 2023, and follow-up data were updated as of September, 2025. The study was reported in accordance with the Strengthening the Reporting of Observational Studies in Epidemiology (STROBE) guidelines.

### Ethical approval

The study protocol was approved by the Bahcesehir University Non-Interventional Ethical Committee (approval number: 2024–06/07, date: 16.04.2025). The study was conducted in accordance with the Declaration of Helsinki. Given the retrospective nature of the study, informed consent was waived. All patient data were anonymized prior to analysis.

### Patient selection

Patients who ≥ 18 years old with histopathologically confirmed WHO grade 2 meningioma who underwent surgical resection followed by RT and had a minimum follow-up of six months were included. Grade 2 meningiomas comprised atypical and chordoid histologic subtypes. Patients with benign (grade 1) or anaplastic (grade 3) meningiomas, multifocal meningiomas, prior cranial RT, synchronous second primary malignancies, or incomplete follow-up data were excluded.

### Definition of treatment groups

Patients were stratified into two groups based on the timing of RT. The early RT group included patients who received adjuvant RT after initial surgery without documented disease progression. The late RT group consisted of patients who were initially managed with surveillance after surgery and subsequently received salvage RT following radiologic or clinical progression, with or without repeat surgical resection.

The interval between surgery and RT in the early RT group was defined as 0–6 months. The median time from surgery to initiation of RT was 1.5 months in the early RT group.

By definition, all patients in the late RT group experienced disease progression prior to RT. Progression events occurring after RT were recorded as a separate variable.

Survival analyses were performed in the entire cohort. Prespecified subgroup analyses were additionally conducted in patients who underwent GTR (Simpson grade 1–3).

### Surgical and pathologic variables

Extent of resection was categorized according to the Simpson grading system. Simpson grades 1–3 were defined as GTR, whereas Simpson grades 4–5 were classified as subtotal resection (STR).

Recorded pathologic variables included histologic subtype (atypical vs chordoid), Ki-67 proliferation index (%), mitotic count (per 10 high-power fields), presence of brain invasion, necrosis, hypercellularity, anaplasia, and TERT promoter mutation status. TERT promoter mutations were evaluated using next-generation sequencing (NGS). However, data were available from only two centers and in selected cases.

### Tumor- and treatment-related variables

Demographic variables (age, sex), clinical characteristics (ECOG (Eastern Cooperative Oncology Group) performance status, antiepileptic and corticosteroid use), and tumor-related features (location, preoperative tumor size) were obtained from medical records and electronic databases.

Tumor location was categorized into three clinically relevant groups: supratentorial non–skull base (convexity and falx/parasagittal tumors), supratentorial skull base (sphenoid wing/parasellar and olfactory groove tumors), and posterior fossa/other locations.

RT-related variables included treatment technique (three-dimensional conformal RT (3D-CRT) or intensity-modulated radiotherapy/volumetric modulated arc therapy (IMRT/VMAT)), total dose (Gy), and number of fractions. Fractionation schedules were classified as conventional (1.8–2.0 Gy per fraction), hypofractionated (>2.4 Gy per fraction), or stereotactic radiosurgery/fractionated stereotactic RT.

### Missing data and multiple imputation

Evaluation of the missing-data profile demonstrated incomplete values for the Ki-67 proliferation index in 107 patients (40.7%), mitotic count in 105 (39.9%), presence of necrosis in 50 (19.0%), and brain invasion in 20 (7.6%). In analyses performed to characterize the missingness mechanism, missingness was significantly associated with diagnostic group (*p* = 0.031), vital status (*p* = 0.005), and surgical extent (*p* = 0.003). These findings rejected the Missing Completely at Random assumption and supported a Missing at Random (MAR) mechanism.

Complete-case analysis would have resulted in substantial information loss, with an estimated 74%–83% reduction in events in OS models, leading to unacceptable loss of statistical power. Therefore, multiple imputation by chained equations (MICE), which yields valid and approximately unbiased estimates under MAR, was used.

The imputation model included age, sex, extent of resection, diagnostic group, total RT dose, fractionation schedule, RT technique, and PFS and OS variables as auxiliary predictors. Continuous variables (Ki-67, mitotic count) were imputed using Bayesian ridge regression, and binary variables (necrosis, brain invasion) were imputed using logistic regression. Ten imputed datasets were generated and combined using Rubin’s rules to obtain pooled estimates. Distributional fidelity was confirmed (Ki-67: original mean 12.6 vs imputed mean 12.4; brain invasion: original 42.0% vs imputed 41.8%).

### Endpoints and definitions

#### The primary endpoints were PFS and OS

In the early RT group, PFS was defined as the interval from the date of surgery to the date of radiologic or clinical progression. In the late RT group, because all patients experienced progression by definition, PFS was calculated from the date of surgery to the initiation of RT. This methodological approach was chosen to establish a comparable time origin between the two groups. Patients without progression were censored at the date of last follow-up.

OS was defined as the interval from the date of surgery to death from any cause. Patients who were alive at last follow-up were censored.

Tumor-related mortality was evaluated as a secondary endpoint. Causes of death were classified as tumor-related or non–tumor-related. Given the long follow-up duration and the inclusion of the COVID-19 pandemic period, a substantial proportion of deaths occurred from non–tumor-related causes, necessitating a competing risk approach.

## Statistical analysis

### Descriptive and comparative analyses

Categorical variables are reported as frequency and percentage. Continuous variables are presented as mean ± standard deviation or median [minimum–maximum], according to distribution. Normality was assessed using the Shapiro–Wilk test and histogram inspection. Between-group comparisons were performed using the χ^2^ test or Fisher exact test for categorical variables, independent-samples t test for normally distributed continuous variables, and the Mann–Whitney U test for non-normally distributed continuous variables.

### Group definitions, surgery, and endpoint definitions

Patients were categorized according to RT timing: early RT after initial surgery (Early RT) versus salvage RT delivered after recurrence (late RT). Extent of resection was classified using the Simpson grading system: Simpson grade 1–3 as GTR and Simpson grade 4–5 as STR.

### Survival and regression modeling

Survival functions were estimated using the Kaplan–Meier method and compared using the log-rank test. Survival rates at 1, 3, 5, and 10 years were reported with 95% confidence intervals (CIs). Median survival was defined at the 50% survival probability; when the survival probability remained > 50% at last follow-up, the median was reported as not reached. Statistical significance was defined as *p* ≤ 0.05 (two-sided).

For prognostic factor assessment, univariable Cox proportional hazards regression was first performed. Variables with *p* < 0.10 in univariable analyses were entered into multivariable models. Model complexity was guided by events per variable (EPV), targeting EPV ≥ 5. When EPV was limited, variables with the largest effect sizes (highest absolute β coefficients) were prioritized. The proportional hazards assumption was evaluated using Schoenfeld residuals and log-minus-log plots. Multicollinearity was assessed using the variance inflation factor (VIF < 5).

### Tumor-related mortality and competing risks

Given the high rate of non–tumor-related deaths, tumor-related mortality was analyzed using two complementary approaches: (1) cause-specific Cox models treating non–tumor-related deaths as censoring events for etiologic interpretation, and (2) Fine–Gray subdistribution hazard models treating non–tumor-related death as a competing event for prognostic assessment and estimation of absolute risk. Cumulative incidence functions (CIFs) were estimated using the Aalen–Johansen method and reported at 1, 3, 5, and 10 years with 95% CIs. Between-group differences in CIFs were assessed using *p* values derived from the Fine–Gray model. In the Fine–Gray framework, tumor-related death was coded as the event of interest (code = 1) and non–tumor-related death as the competing event (code = 2). Results from both approaches were interpreted comparatively.

Analyses were conducted in the overall cohort (*n* = 263) and separately in the prespecified GTR subgroup (*n* = 162). Outcomes included 1-, 3-, and 10-year PFS and OS, as well as tumor-specific survival.

Statistical analyses were performed using Jamovi 2.6.44, JASP 0.95.4.0, Python 3.11 (lifelines 0.27.8, pandas, numpy, matplotlib), and R 4.5.2 (survival, cmprsk, haven). A two-sided *p* ≤ 0.05 was considered statistically significant.

## Results

### Kaplan–Meier survival analyses

#### Survival outcomes in the overall cohort

**Fig. 1:**
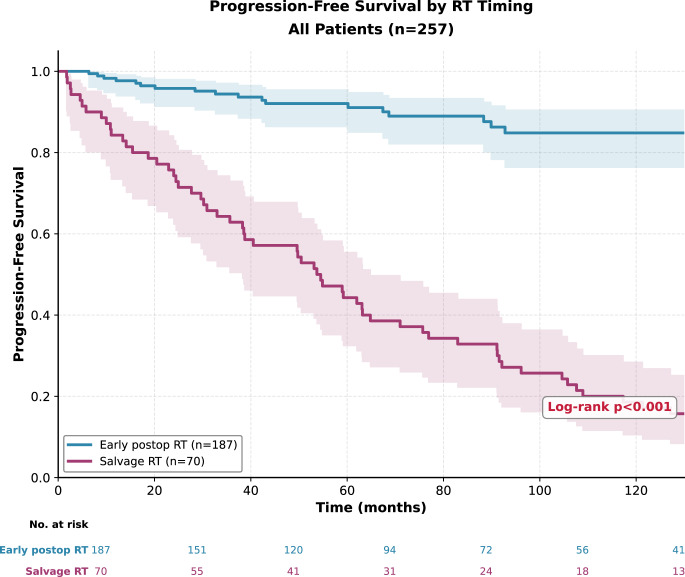
Progression-free survival according to radiotherapy timing in the overall cohort (*n* = 257). Patients receiving early postoperative radiotherapy demonstrated significantly longer progression-free survival compared with those treated with radiotherapy at recurrence (log-rank *p* < 0.001)

The median duration of follow-up was 84.6 months. PFS analyses by RT timing were performed in 257 patients. In the early RT group (*n* = 187), 18 progression events were observed, whereas in the late RT group (*n* = 70), all patients experienced progression during follow-up. Median PFS was not reached in the early RT group and was 53.7 months in the late RT group. One-year PFS rates were 98.3% in the early RT group and 84.3% in the late group. Ten-year PFS was 84.8% in the early RT group but declined to 18.6% in the late RT group (*p* < 0.001) (Table 1; Fig. 1).

**Table 1:** Survival outcomes by radiotherapy timing in the overall cohort

Outcome / Treatment Group	n	Event	Median (month)	1-Year survival %, (95% CI)	3-Year survival %, (95% CI)	5-Year survival %, (95% CI)	10-Year survival %, (95% CI)	p
**Progression-free survival**								**<0,001**
Early RT	187	18	NR	98,3 (94,7–99,4)	94,4 (89,5–97,1)	92,1 (86,4–95,4)	84,8 (76,3–90,5)	
Late RT	70	70	53,7	84,3 (73,4–91,0)	62,9 (50,4–73,0)	44,3 (32,5–55,4)	18,6 (10,5–28,4)	
Total	257	88	147,6	94,3 (90,6–96,6)	84,8 (79,4–88,9)	76,1 (69,7–81,4)	58,0 (49,7–65,4)	
**Overal survival**								0,175
Early RT	187	34	NR	98,9 (95,7–99,7)	95,1 (90,4–97,5)	90,1 (83,7–94,0)	75,0 (65,1–82,5)	
Late RT	70	18	NR	100 (–)	97,1 (88,8–99,3)	94,0 (84,9–97,7)	85,5 (73,8–92,2)	
Total	257	52	NR	99,2 (96,9–99,8)	95,7 (92,1–97,6)	91,4 (86,6–94,5)	79,1 (71,9–84,6)	

**Bolded** *p* values indicate statistical significance (*p* ≤ 0.05). Survival rates were estimated using the Kaplan–Meier method, with 95% confidence intervals shown in parentheses. Group comparisons were performed using the log-rank test. NR: Not reached, RT:radiotherapy

A different pattern was observed for overall survival (OS). Median OS was not reached in either group. Ten-year OS was 75.0% in the early RT group and 85.5% in the late RT group. Although OS was numerically higher in the late group, this difference was not statistically significant (*p* = 0.175) (Table 1).

### Survival outcomes in the Simpson grade 1–3 (gross total resection) subgroup

**Fig. 2:**
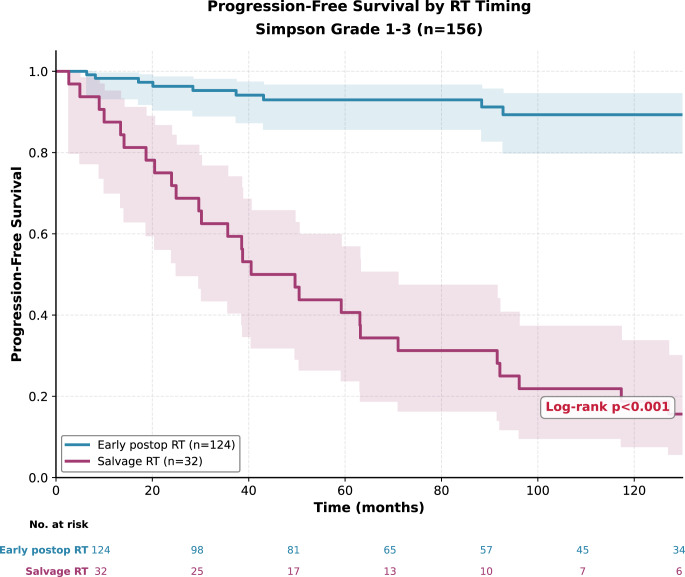
Progression-free survival according to radiotherapy timing in patients who underwent gross total resection (Simpson grade 1–3; *n* = 156). Early postoperative radiotherapy was associated with significantly longer progression-free survival compared with radiotherapy delivered at recurrence (log-rank *p* < 0.001)

In patients who underwent gross total resection, PFS analyses were conducted in 156 patients. In the early RT group (newly diagnosed WHO grade 2 meningoma, *n* = 124), only nine progression events occurred, whereas all patients in the late RT group (recurrent WHO grade 2 meningioma, *n* = 32) experienced progression during follow-up. Median PFS was not reached in the early RT group and was 40.5 months in the late group. Ten-year PFS remained high in the early RT group (89.3%) but decreased to 18.8% in the late RT group (*p* < 0.001) (Table 2; Fig. 2).

OS in this subgroup showed a similar pattern to that observed in the overall cohort. Ten-year OS was 79.0% in the early RT group and 81.4% in the late group, with no statistically significant difference between groups (*p* = 0.719) (Table 2).

**Table 2:** Survival outcomes according to radiotherapy timing after gross total resection (simpson grade 1–3)

Outcome / Treatment Group	n	Event	Median (month)	1-Year survival %, (95% CI)	3-Year survival %, (95% CI)	5-Year survival %, (95% CI)	10-Year survival %, (95% CI)	p
**Progression-free survival**								**<0,001**
Early RT	124	9	NR	98,3 (93,3–99,6)	95,3 (89,0–98,0)	93,0 (85,8–96,6)	89,3 (79,9–94,5)	
Late RT	32	32	40,5	87,5 (70,0–95,1)	59,4 (40,5–74,0)	40,6 (23,8–56,8)	18,8 (7,6–33,7)	
Total	156	41	186,6	95,9 (91,2–98,2)	86,8 (79,8–91,5)	79,7 (71,5–85,8)	68,5 (58,3–76,7)	
**Overal survival**								0,719
Early RT	124	19	NR	100 (–)	97,9 (92,0–99,5)	94,0 (86,1–97,5)	79,0 (66,6–87,2)	
Late RT	32	7	NR	100 (–)	96,8 (79,2–99,5)	96,8 (79,2–99,5)	81,4 (60,7–91,8)	
Total	156	26	NR	100 (–)	97,7 (92,9–99,2)	94,8 (88,8–97,6)	80,0 (70,1–86,9)	

**Bolded** *p* values indicate statistical significance (*p* ≤ 0.05). Survival rates were estimated using the Kaplan–Meier method, with 95% confidence intervals shown in parentheses. Group comparisons were performed using the log-rank test. NR: Not reached, RT:radiotherapy

### Overall cohort and comparison by extent of resection

Among 263 patients, 162 (61.6%) underwent gross total resection (GTR; Simpson grade 1–3) and 101 (38.4%) underwent subtotal resection (STR; Simpson grade 4–5). Mean age was 53.8 ± 13.6 years, and 57.8% were female. On pathologic review, 95.8% of tumors were classified as atypical and 4.2% as chordoid meningioma. The median Ki-67 proliferation index was 10%, and the median mitotic count was 5 per 10 high-power fields (Table 3).

**Table 3:** Comparison of patient, tumor, treatment, and outcome characteristics according to extent of surgical resection

Variables		Total (n = 263)	Simpson 1–3 (n = 162)	Simpson 4–5 (n = 101)	p
Age *(year)*		53,8 ± 13,6	53,8 ± 12,9	53,8 ± 14,6	0,997
Age group	≤50	107 (40,7)	68 (42,0)	39 (38,6)	0,681
	>50	156 (59,3)	94 (58,0)	62 (61,4)	
Gender	Female	152 (57,8)	93 (57,4)	59 (58,4)	0,974
	Male	111 (42,2)	69 (42,6)	42 (41,6)	
ECOG Performance Score	0	124 (49,0)	85 (54,1)	39 (40,6)	0,128
	1	102 (40,3)	59 (37,6)	43 (44,8)	
	2	22 (8,7)	11 (7,0)	11 (11,5)	
	3	5 (2,0)	2 (1,3)	3 (3,1)	
Tumor size *(mm)*		45,0 [13,0–122,0]	44,0 [13,0–120,0]	45,0 [15,0–122,0]	0,266
Tumor Location	Convexity	88 (33,7)	68 (42,2)	20 (20,0)	**<0,001**
	Falx/Parasagittal	62 (23,8)	45 (28,0)	17 (17,0)	
	Sphenoidwing/Parasellar	52 (19,9)	13 (8,1)	39 (39,0)	
	Olfactory Sulcus	15 (5,7)	12 (7,5)	3 (3,0)	
	Posterior Fossa	9 (3,4)	6 (3,7)	3 (3,0)	
	Other	35 (13,4)	17 (10,6)	18 (18,0)	
Pathology	Atypical	252 (95,8)	156 (96,3)	96 (95,0)	0,754
	Chordoid	11 (4,2)	6 (3,7)	5 (5,0)	
Ki-67 Proliferation Index (%)		10,0 [1,0–75,0]	10,0 [1,0–75,0]	10,0 [1,0–30,0]	0,835
Mitosis *(/10 HPF)*		5,0 [0,0–57,0]	5,0 [2,0–57,0]	4,0 [0,0–18,0]	0,203
Brain Invasion, *yes*		141 (58,0)	104 (69,3)	37 (39,8)	**<0,001**
TERT mutation	No	66 (28,1)	48 (32,4)	18 (20,7)	0,098
	Yes	4 (1,7)	3 (2,0)	1 (1,1)	
	Unknown	165 (70,2)	97 (65,5)	68 (78,2)	
Anaplasia, *yes*		6 (2,8)	3 (2,1)	3 (4,1)	0,417
Hypercellularity, *yes*		109 (53,4)	71 (51,8)	38 (56,7)	0,611
Necrosis, *yes*		146 (68,5)	94 (66,7)	52 (72,2)	0,503
RT Technique	3D-CRT	53 (20,2)	32 (19,8)	21 (20,8)	0,963
	IMRT/VMAT	210 (79,8)	130 (80,2)	80 (79,2)	
Total Dose *(Gy)*		54,0 [12,0–66,0]	54,0 [12,0–66,0]	54,0 [15,0–66,0]	0,066
Fraction number		30,0 [1,0–33,0]	30,0 [1,0–33,0]	30,0 [1,0–33,0]	**0,008**
RT fractionation	Conventional	198 (75,3)	132 (81,5)	66 (65,3)	**0,008**
	Hypofractionated	41 (15,6)	17 (10,5)	24 (23,8)	
	SRS/fSRS	24 (9,1)	13 (8,0)	11 (10,9)	
RT timing	Early RT	192 (73,0)	129 (79,6)	63 (62,4)	**0,003**
	Late RT	71 (27,0)	33 (20,4)	38 (37,6)	
Antiepileptic use, *yes*		114 (53,0)	72 (50,7)	42 (57,5)	0,420
Steroid use, *yes*		140 (71,8)	98 (73,7)	42 (67,7)	0,492
1. year progression, *yes*		16 (6,4)	9 (5,9)	7 (7,1)	0,904
3. year progression, *yes*		37 (17,5)	22 (17,5)	15 (17,6)	0,999
10. year progression, *yes*		78 (58,2)	38 (48,7)	40 (71,4)	**0,014**
Recurrence, *yes*		172 (65,4)	118 (72,8)	54 (53,5)	**0,002**
Cause of death	Non-tumoral	30 (56,6)	19 (70,4)	11 (42,3)	0,075
	Tumor	23 (43,4)	8 (29,6)	15 (57,7)	
Disease related death, *yes*		23 (9,9)	8 (5,6)	15 (16,7)	**0,011**
Death From Any Cause, *yes*		53 (20,2)	27 (16,7)	26 (25,7)	0,104
Salvage therapy	Surgery	25 (46,3)	12 (48,0)	13 (44,8)	0,662
	Surgery + RT	13 (24,1)	7 (28,0)	6 (20,7)	
	Only RT	16 (29,6)	6 (24,0)	10 (34,5)	

**Bolded** *p* values indicate statistical significance (*p* ≤ 0.05). Continuous variables are presented as mean ± standard deviation for normally distributed data and median [minimum–maximum] for non–normally distributed data. Categorical variables are reported as n (%). PFS, progression-free survival; OS, overall survival; ECOG, Eastern Cooperative Oncology Group; HPF, high-power field; TERT, telomerase reverse transcriptase; 3D-CRT, three-dimensional conformal radiotherapy; IMRT, intensity-modulated radiotherapy; VMAT, volumetric modulated arc therapy; SRS, stereotactic radiosurgery; fSRS, fractionated stereotactic radiosurgery; RT, radiotherapy.

When stratified by extent of resection, there were no significant differences between the GTR and STR groups in age, sex, ECOG performance status, preoperative tumor size, histologic subtype, or Ki-67 index (all *p* > 0.05). In contrast, tumor location differed markedly (*p* < 0.001): GTR was more frequent for convexity and falx/parasagittal tumors, whereas STR was performed in the majority of sphenoid wing/parasellar tumors (75.0%). Brain invasion was significantly more common in the GTR group than in the STR group (69.3% vs 39.8%; *p* < 0.001).

RT characteristics also differed by surgical extent. Conventional fractionation was used more frequently in the GTR group than in the STR group (81.5% vs 65.3%; *p* = 0.008), and early adjuvant RT was more commonly delivered after GTR (79.6% vs 62.4%; *p* = 0.003).

With respect to outcomes, the 10-year progression rate was significantly higher after STR than after GTR (71.4% vs 48.7%; *p* = 0.014). Tumor-related mortality was approximately threefold higher in the STR group (16.7% vs 5.6%; *p* = 0.011). All-cause mortality was numerically higher in the STR group, but did not reach statistical significance (25.7% vs 16.7%; *p* = 0.104). Salvage treatment modalities did not differ between groups (*p* = 0.662).

### GTR subgroup: early RT versus late RT

Among the 162 patients treated with GTR, 129 (79.6%) received early adjuvant RT and 33 (20.4%) received late salvage RT. Age, sex distribution, and ECOG performance status were similar between groups (all *p* > 0.05). Tumor location differed (*p* = 0.046), with convexity tumors more frequently treated with early RT (47.7% vs 21.2%). Simpson grade distribution also differed significantly (*p* = 0.009): Simpson grade 2 resections were more common in the early RT group (38.0% vs 12.1%), whereas Simpson grade 3 resections were more common in the late RT group (36.4% vs 18.6%) (Table 4).

**Table 4:** Comparison by RT timing after gross total resection (simpson grade 1–3)

Variables		Early RT (n = 129)	Late RT (n = 33)	p
Age *(year)*		53,7 ± 12,7	54,0 ± 13,9	0,921
Age group	≤50 yaş	55 (42,6)	13 (39,4)	0,889
	>50 yaş	74 (57,4)	20 (60,6)	
Gender	Female	72 (55,8)	21 (63,6)	0,539
	Male	57 (44,2)	12 (36,4)	
ECOG Performance Score	0	73 (57,9)	12 (38,7)	0,112
	1	43 (34,1)	16 (51,6)	
	2	9 (7,1)	2 (6,5)	
	3	1 (0,8)	1 (3,2)	
Tumor size *(mm)*		45,0 [16,0–90,0]	37,0 [13,0–120,0]	0,093
Tumor Location	Convexity	61 (47,7)	7 (21,2)	**0,046**
	Falx/Parasagittal	33 (25,8)	12 (36,4)	
	Sphenoidwing/Parasellar	8 (6,2)	5 (15,2)	
	Olfactory Sulcus	10 (7,8)	2 (6,1)	
	Posterior Fossa	4 (3,1)	2 (6,1)	
	Other	12 (9,4)	5 (15,2)	
Simpson grade	Grade 1	56 (43,4)	17 (51,5)	**0,009**
	Grade 2	49 (38,0)	4 (12,1)	
	Grade 3	24 (18,6)	12 (36,4)	
Pathology	Atypical	124 (96,1)	32 (97,0)	0,999
	Chordoid	5 (3,9)	1 (3,0)	
Ki-67 Proliferation Index (%)		10,0 [1,0–75,0]	11,0 [2,0–45,0]	0,639
Mitosis *(/10 HPF)*		5,0 [2,0–57,0]	9,0 [2,0–30,0]	**0,005**
Brain Invasion, *yes*		84 (70,0)	20 (66,7)	0,894
TERT Mutation	Yes	31 (26,3)	17 (56,7)	**0,001**
	No	1 (0,8)	2 (6,7)	
	Unknown	86 (72,9)	11 (36,7)	
Anaplasia, *yes*		3 (2,6)	0 (0,0)	0,999
Hypercellularity, *yes*		54 (49,1)	17 (63,0)	0,281
Necrosis, *yes*		76 (67,3)	18 (64,3)	0,941
RT technique	3D-CRT	26 (20,2)	6 (18,2)	0,993
	IMRT/VMAT	103 (79,8)	27 (81,8)	
Total Dose *(Gy)*		54,0 [12,0–60,0]	36,0 [13,0–66,0]	**<0,001**
Fraction number		30,0 [1,0–33,0]	10,0 [1,0–33,0]	**<0,001**
RT fractionation	Conventional	119 (92,2)	13 (39,4)	**<0,001**
	Hypofractionated	4 (3,1)	13 (39,4)	
	SRS/fSRS	6 (4,7)	7 (21,2)	
Steroid use, *yes*		89 (80,2)	9 (40,9)	**<0,001**
Antiepileptic use, *yes*		60 (51,3)	10 (40,0)	0,421
1. year progression, *yes*		3 (2,5)	6 (18,2)	**0,004**
3. year progression, *yes*		7 (7,5)	15 (45,5)	**<0,001**
10. year progression, *yes*		11 (24,4)	27 (81,8)	**<0,001**
Cause of death	Non-tumoral	16 (84,2)	3 (37,5)	**0,027**
	Tumor	3 (15,8)	5 (62,5)	
Disease related death, *yes*		3 (2,7)	5 (16,7)	**0,011**
Death From Any Cause, *yes*		19 (14,7)	8 (24,2)	0,198
Salvage therapy	Surgery	6 (54,5)	6 (42,9)	0,145
	Surgery + RT	1 (9,1)	6 (42,9)	
	Only RT	4 (36,4)	2 (14,3)	

**Bolded** *p* values indicate statistical significance (*p* ≤ 0.05). Continuous variables are presented as mean ± standard deviation for normally distributed data and median [minimum–maximum] for non–normally distributed data. Categorical variables are reported as n (%). PFS, progression-free survival; OS, overall survival; ECOG, Eastern Cooperative Oncology Group; HPF, high-power field; TERT, telomerase reverse transcriptase; 3D-CRT, three-dimensional conformal radiotherapy; IMRT, intensity-modulated radiotherapy; VMAT, volumetric modulated arc therapy; SRS, stereotactic radiosurgery; fSRS, fractionated stereotactic radiosurgery; RT, radiotherapy.

Regarding pathologic characteristics, the late RT group demonstrated a higher median mitotic count than the early RT group (9 vs 5 per 10 high-power fields; *p* = 0.005). TERT mutation status also differed (*p* = 0.001), with a higher proportion of patients having known TERT status in the late RT group. RT parameters were substantially different between groups: the median total dose was 54 Gy in the early RT group versus 36 Gy in the late group (*p* < 0.001), and conventional fractionation predominated in the early RT group (92.2% vs 39.4%; *p* < 0.001).

Progression rates were consistently higher in the late RT group at all assessed time points: 1-year progression, 18.2% vs 2.5% (*p* = 0.004); 3-year progression, 45.5% vs 7.5% (*p* < 0.001); and 10-year progression, 81.8% vs 24.4% (*p* < 0.001). Tumor-related mortality was approximately sixfold higher in the late RT group (16.7% vs 2.7%; *p* = 0.011), whereas all-cause mortality did not differ significantly (24.2% vs 14.7%; *p* = 0.198).

### GTR subgroup: recurrence versus no recurrence

When patients treated with GTR were stratified by recurrence status, 118 (72.8%) did not develop recurrence during follow-up and 44 (27.2%) experienced recurrence. Recurrence was defined as radiologically confirmed disease progression during the course of follow-up. Demographic characteristics and preoperative tumor size were comparable (*p* > 0.05). Simpson grade distribution differed significantly (*p* = 0.002): Simpson grade 2 resections were more frequent among nonrecurrent patients (39.0% vs 15.9%), whereas Simpson grade 3 resections were more common among patients who recurred (38.6% vs 16.1%) (Supplemantary Table-1).

Among pathologic variables, mitotic count was higher in the recurrence group (median 8 vs 4.5 per 10 high-power fields; *p* = 0.004). TERT mutation status differed between groups (*p* = 0.002), with higher availability of TERT status in the recurrence group. RT characteristics also differed: median total dose was lower in the recurrence group (51 vs 54 Gy; *p* < 0.001), conventional fractionation was less common (50.0% vs 93.2%; *p* < 0.001), and hypofractionated treatment was more frequent (31.8% vs 2.5%). By definition of treatment strategy, all nonrecurrent patients received early adjuvant RT, whereas approximately three-quarters of recurrent patients received late RT (*p* < 0.001). No tumor-related deaths were observed in the nonrecurrent group, compared with 19.5% in the recurrence group (*p* < 0.001).

### Overall survival (overall cohort)

For overall survival, 210 patients (79.8%) were alive and 53 (20.2%) had died at last follow-up. Age was strongly associated with mortality, with decedents being older (60.8±13.4 vs 52.0 ± 13.1 years; *p* < 0.001). The proportion of patients aged > 50 years was higher among decedents (79.2% vs 54.3%; *p* = 0.001). Male sex was also more frequent among decedents (54.7% vs 39.0%; *p* = 0.039). Extent of resection did not differ significantly between survivors and decedents (*p* = 0.074) (Supplemantary Table 2).

Brain invasion was more common among decedents than survivors (57.4% vs 38.3%; *p* = 0.017). RT technique was associated with mortality: 3-dimensional conformal RT (3D-CRT) was used more frequently in decedents (34.0% vs 16.7%; *p* = 0.005). Total dose, number of fractions, and fractionation schedule did not differ significantly (all *p* > 0.05).

### Results in the GTR subgroup (selected endpoints)

In the GTR subgroup (*n* = 162), 135 patients (83.3%) were alive and 27 (16.7%) had died. Decedents were older (60.6±11.5 vs 52.4 ± 12.8 years; *p* = 0.002). Sex, ECOG performance status, tumor location, and Simpson grade did not differ significantly between survivors and decedents (all *p* > 0.05). RT technique remained strongly associated with mortality (*p* < 0.001), with 3D-CRT used approximately threefold more frequently among decedents (44.4% vs 14.8%) (Supplemantary Table 3).

### Cox regression and competing-risk analyses

#### One-year progression-free survival

Cox regression analysis evaluating factors associated with 1-year progression-free survival (PFS) included 249 patients and 14 events. In univariable analysis, only treatment group was statistically significant, with late RT associated with a tenfold higher risk of progression (HR = 10.20; 95% CI, 2.84–36.57; *p* < 0.001). In the multivariable model, this effect was further accentuated, and late RT was confirmed as an independent prognostic factor (adjusted HR [aHR]=12.56; 95% CI, 3.32–47.56; *p* < 0.001). Presence of necrosis and RT fractionation schedule were included in the model but did not reach statistical significance. Model discrimination was high (C-index = 0.803); however, the global Schoenfeld test indicated a borderline violation of the proportional hazards assumption (*p* = 0.034). This finding may be attributable to the low number of events (*n* = 14) and short follow-up duration, and results should be interpreted accordingly.

#### Three-year progression-free survival

The 3-year PFS analysis included 209 patients and 35 events. In univariable analysis, treatment group (HR = 7.09; *p* < 0.001), mitotic count (HR = 1.04; *p* = 0.010), and hypofractionated RT (HR = 2.11; *p* = 0.041) were statistically significant. In the multivariable model, late RT emerged as the strongest independent prognostic factor (aHR = 7.52; 95% CI, 3.35–16.90; *p* < 0.001), and mitotic count remained independently associated with progression (aHR = 1.05; 95% CI, 1.00–1.09; *p* = 0.046). Hypofractionated RT lost statistical significance after adjustment for treatment group, consistent with confounding. Model fit was good (C-index = 0.772), and the proportional hazards assumption was satisfied (Schoenfeld *p* = 0.810).

#### Ten-year progression-free survival

For 10-year PFS, a standard Cox model was constructed including 133 patients and 77 events. In univariable analysis, extent of resection (HR = 1.66; *p* = 0.026), treatment group (HR = 4.05; *p* < 0.001), Ki-67 index (HR = 1.04; *p* = 0.008), mitotic count (HR = 1.06; *p* = 0.006), SRS/fSRS (HR = 1.88; *p* = 0.046), and IMRT/VMAT technique (HR = 1.94; *p* = 0.014) were statistically significant. In the multivariable model, only late RT remained an independent prognostic factor (aHR = 3.31; 95% CI, 1.81–6.07; *p* < 0.001). Overall model performance was acceptable (C-index = 0.697); however, violations of the proportional hazards assumption were detected for Ki-67 (*p* = 0.014) and mitotic count (*p* = 0.037) (Table 5).

**Table 5:** Prognostic factors for 10-Year progression-free survival in the overall cohort: Cox regression analysis (*n* = 133)

Variables	Univariate		Multivariate	
	HR [%95 CI]	p	aHR [%95 CI]	p
Age *(year)*	1,01 [0,99–1,03]	0,261	—	—
Gender				
Female *(ref)*	1	—	—	—
Male	1,25 [0,80–1,97]	0,331	—	—
Extent of surgical resection				
Simpson grade 1–3 *(ref)*	1	—	1	—
Simpson grade 4–5	1,66 [1,06–2,60]	**0,026**	1,32 [0,80–2,18]	0,276
RT timing				
Early RT *(ref)*	1	—	1	—
Late RT	4,05 [2,42–6,78]	**<0,001**	3,31 [1,81–6,07]	**<0,001**
Tumor location				
Supratentorial Non-Base *(ref)*	1	—	—	—
Supratentorial Skull Base	0,92 [0,55–1,53]	0,748	—	—
Posterior Fossa/Other	1,05 [0,61–1,80]	0,859	—	—
Ki-67 Proliferation Index (%)	1,04 [1,01–1,08]	**0,008**	1,03 [0,99–1,08]	0,16
Mitosis *(/10 BBA)*	1,06 [1,02–1,11]	**0,006**	1,02 [0,95–1,10]	0,609
Necrosis, *yes*	0,89 [0,53–1,49]	0,65	—	—
Brain Invasion, *yes*	1,44 [0,92–2,26]	0,109	—	—
Total Dose *(Gy)*	0,98 [0,97–1,00]	0,063	1,00 [0,98–1,04]	0,747
RT Fractionation				
Conventional *(ref)*	1	—	1	—
Hypofractionated	1,24 [0,76–2,00]	0,387	—	—
SRS/fSRS	1,88 [1,01–3,48]	**0,046**	1,41 [0,57–3,46]	0,454
RT Technique				
3D-CRT *(ref)*	1	—	1	—
IMRT/VMAT	1,94 [1,14–3,29]	**0,014**	1,20 [0,64–2,24]	0,563

Model statistics: number of events = 77; C-index = 0.697; likelihood ratio test: χ^2^ = 39.76, *p* < 0.001; global Schoenfeld test: χ^2^ = 15.50, df = 7, *p* = 0.030. Bolded *p* values indicate statistical significance (*p* ≤ 0.05). Variables with *p* < 0.10 in univariable analyses, as well as clinically relevant variables, were included in the multivariable model. Violations of the proportional hazards assumption were identified for Ki-67 (*p* = 0.014) and mitotic count (*p* = 0.037). HR, hazard ratio; aHR, adjusted hazard ratio; CI, confidence interval; RT, radiotherapy; HPF, high-power field; 3D-CRT, three-dimensional conformal radiotherapy; IMRT, intensity-modulated radiotherapy; VMAT, volumetric modulated arc therapy; SRS, stereotactic radiosurgery; fSRS, fractionated stereotactic radiosurgery.

#### Time-dependent Cox modeling

Given the violation of the proportional hazards assumption, an extended Cox model incorporating time-dependent covariates was constructed. Interaction terms for time×Ki-67 and time×mitotic count were included. In this extended model, none of the covariates reached statistical significance; both the Ki-67×log(time) (*p* = 0.191) and mitotic count×log(time) (*p* = 0.371) interactions were nonsignificant, indicating no definitive evidence that the effects of these pathologic markers varied over time. Notably, the extended model demonstrated a substantial improvement in discrimination (C-index increased from 0.697 to 0.938), and likelihood ratio testing confirmed a significant improvement in model fit (χ^2^ = 39.27; *p* < 0.001), suggesting that incorporation of time-dependent covariates enhanced overall model performance.

#### All-cause mortality

Cox regression analysis for all-cause mortality included 263 patients and 53 deaths. In univariable analysis, older age (HR = 1.07; *p* < 0.001), male sex (HR = 2.16; *p* = 0.006), and Ki-67 index (HR = 1.04; *p* = 0.050) were statistically significant. In the multivariable model, three independent prognostic factors were identified: older age (aHR = 1.07; 95% CI, 1.04–1.09; *p* < 0.001), male sex (aHR = 1.83; 95% CI, 1.05–3.21; *p* = 0.035), and subtotal resection (aHR = 1.88; 95% CI, 1.06–3.36; *p* = 0.032). Although extent of resection was not significant in univariable analysis, it emerged as an independent factor in the multivariable model, suggesting negative confounding by age and sex. Treatment group was not significantly associated with all-cause mortality (aHR = 0.67; *p* = 0.221). Model performance was adequate (C-index = 0.737), and the proportional hazards assumption was satisfied (Schoenfeld *p* = 0.423) (Supplemantary Table 4).

#### Tumor-related mortality: cause-specific and competing-risk analyses

Cause-specific hazard analysis for tumor-related death censored 30 patients who died from non–tumor-related causes and included 23 tumor-related deaths. In the multivariable cause-specific model, older age (adjusted cause-specific HR [acsHR]=1.04; *p* = 0.049), male sex (acsHR = 2.90; *p* = 0.016), and mitotic count (acsHR = 1.06; *p* = 0.020) were independent prognostic factors. Extent of resection demonstrated borderline significance (acsHR = 2.49; *p* = 0.060) (Supplemantary Table 5).

A Fine–Gray subdistribution hazard model was subsequently constructed to account for competing risks. In the multivariable model, male sex (adjusted subdistribution HR [asHR]=2.70; *p* = 0.024), subtotal resection (asHR = 2.55; *p* = 0.044), and mitotic count (asHR = 1.06; *p* = 0.019) remained independent predictors of tumor-related death. late RT showed borderline significance (asHR = 2.43; *p* = 0.052). Age, which was significant in the cause-specific model (*p* = 0.049), was no longer significant in the subdistribution model (*p* = 0.634), likely reflecting its association with both tumor-related and competing mortality risks (Supplemantary Table 6).

#### Cumulative incidence of tumor-related death

Cumulative incidence of tumor-related death according to RT timing was estimated using the Aalen–Johansen method. At 10 years, cumulative incidence was 4.8% (95% CI, 1.9–9.7) in the early RT group and 7.6% (95% CI, 2.8–15.5) in the late RT group, with a statistically significant difference between groups (*p* = 0.021) (Supplemantary Table 7; Supplemantary Figure-1).

#### Gross total resection subgroup analyses

Among patients undergoing gross total resection, the 1-year PFS analysis included 151 patients and only seven events. In the multivariable model, no covariate reached statistical significance. Given the low event count and inadequate events-per-variable ratio (EPV = 3.5 < 10), results should be interpreted with caution; the proportional hazards assumption was also violated (Schoenfeld *p* = 0.038).

In the 3-year analysis (125 patients, 20 events), late RT was the only independent prognostic factor (aHR = 4.26; *p* = 0.013). RT fractionation lost significance after adjustment for treatment group, consistent with confounding.

In the 10-year analysis (77 patients, 37 events), male sex (aHR = 3.89; *p* = 0.001) and late RT (aHR = 4.01; *p* = 0.002) were identified as independent prognostic factors (Table 6).

**Table 6:** Prognostic factors for 10-Year progression-free survival after gross total resection (simpson grade 1–3): Cox regression analysis (*n* = 77))

Variables	Univariate		Multivariate	
	HR [%95 CI]	p	aHR [%95 CI]	p
**Age** *(year)*	1,00 [0,98–1,03]	0,764	—	—
**Gender**				
Female *(ref)*	1	—	1	—
Male	2,18 [1,14–4,18]	**0,019**	3,89 [1,72–8,80]	**0,001**
**RT timing**				
Early RT *(ref)*	1	—	1	—
Late RT	5,23 [2,56–10,71]	**<0,001**	4,01 [1,68–9,55]	**0,002**
**Tumor location**				
Supratentorial Non-Base *(ref)*	1	—	—	—
Supratentorial Skull Base	1,31 [0,55–3,15]	0,543	—	—
Posterior Fossa/Other	1,04 [0,46–2,37]	0,92	—	—
**Ki-67 Proliferation Index (%)**	1,05 [1,01–1,08]	**0,019**	—	—
**Mitosis** *(/10 BBA)*	1,08 [1,04–1,13]	**<0,001**	1,04 [0,98–1,11]	0,212
**Necrosis**, ***yes***	1,12 [0,57–2,21]	0,738	—	—
**Brain Invasion**, ***yes***	2,02 [1,04–3,94]	**0,038**	—	—
**Total Dose** *(Gy)*	0,97 [0,95–0,99]	**0,002**	1,00 [0,93–1,08]	0,918
**RT Fractionation**				
Conventional *(ref)*	1	—	1	—
Hypofractionated	2,13 [1,05–4,33]	**0,036**	2,24 [0,36–13,84]	0,385
SRS/fSRS	2,70 [1,18–6,17]	**0,018**	1,55 [0,09–27,18]	0,763
**RT Technique**				
3D-CRT *(ref)*	1	—	—	—
IMRT/VMAT	2,21 [0,97–5,03]	0,06	—	—

Model statistics: number of events = 37; C-index = 0.765; likelihood ratio test: χ^2^ = 37.23, *p* < 0.001; global Schoenfeld test: χ^2^ = 10.31, df = 6, *p* = 0.112. Bolded *p* values indicate statistical significance (*p* ≤ 0.05). In the multivariable model, male sex (aHR = 3.89) and Late radiotherapy (aHR = 4.01) were identified as independent prognostic factors. Variables that were significant in univariable analysis—including mitotic count, Ki-67, brain invasion, and radiotherapy fractionation schedule—did not retain statistical significance in the multivariable model. HR, hazard ratio; aHR, adjusted hazard ratio; CI, confidence interval; RT, radiotherapy; HPF, high-power field; 3D-CRT, three-dimensional conformal radiotherapy; IMRT, intensity-modulated radiotherapy; VMAT, volumetric modulated arc therapy; SRS, stereotactic radiosurgery; fSRS, fractionated stereotactic radiosurgery.

For all-cause mortality in the gross total resection cohort (162 patients, 27 deaths), older age (aHR = 1.06; *p* < 0.001) and male sex (aHR = 2.37; *p* = 0.033) remained independent predictors. Treatment group was not significantly associated with overall survival (Supplemantary Table 8).

Cause-specific and Fine–Gray analyses for tumor-related death in the gross total resection cohort were limited by a very low number of events (*n* = 8), and multivariable results should therefore be considered exploratory (Supplemantary Tables 9–10). The 10-year cumulative incidence of tumor-related death was 2.4% in the early RT group and 10.6% in the late RT group, representing a statistically significant difference (*p* = 0.039). In patients undergoing gross total resection, early RT was associated with a more than fourfold reduction in the 10-year risk of tumor-related death (Supplemantary Table-11; Supplemantary Figure 2).

## Discussion

This multicenter retrospective cohort study demonstrates a marked progression-free survival advantage of adjuvant RT compared with late RT in the management of WHO grade 2 meningiomas. Among patients who underwent Simpson grade 1–3 resection, the 3-year PFS rate was 92.5% in the early RT group versus 61.3% in the late RT group, representing a highly statistically significant difference. With extended follow-up to 10 years, this disparity became even more pronounced: PFS was maintained in 89.3% of patients receiving early RT, whereas durable disease control was observed in only 18.2% of those treated with late RT.

The findings of the present study are largely consistent with the results of contemporary meta-analyses in the literature. In a comprehensive meta-analysis including 2,904 patients, Chun et al. demonstrated that adjuvant RT significantly reduced the risk of recurrence (odds ratio, 0.50; *p* < 0.0001) and was consistently associated with higher PFS at 1, 3, and 5 years compared with observation alone [16]. Similarly, a meta-analysis by Verly et al., encompassing 3,822 patients, showed that the addition of RT after GTR resulted in a significant improvement in PFS (hazard ratio, 0.849; *p* = 0.035) [17].

In addition, the 2- and 5-year progression-free survival rates reported by Unterberger et al. (100% and 70.6% with adjuvant RT vs 69% and 39.2% with surgery alone; *p* = 0.004) closely parallel the outcomes observed in our cohort [18]. However, the 3-year PFS rate of 92.5% observed in our cohort exceeds most values reported in the existing literature. Potential explanations for this discrepancy include the fact that all patients in our series received RT, the predominant use of contemporary RT techniques (IMRT/VMAT in 80.2% of cases), and the systematic assessment of surgical extent based primarily on postoperative MRI, which may have enabled more accurate classification of resection status.

Conversely, several studies have reported no significant benefit associated with adjuvant RT. In a multicenter series of 258 patients, Keric et al. found that RT did not improve PFS, identifying Simpson grade and age as the most relevant prognostic factors [19]. Potential explanations for these discrepant findings include differences in patient selection, duration of follow-up, and timing of RT. In particular, in retrospective analyses, the preferential allocation of higher-risk patients to RT may introduce selection bias, thereby confounding observed treatment effects.

Despite the marked improvement in progression-free survival observed in our cohort, no significant difference in OS was detected, a finding that is consistent with the existing literature. Meta-analyses by Chun et al. and Verly et al. similarly demonstrated that although adjuvant RT improves PFS, its effect on OS is either absent or limited to a non-significant trend (HR 0.79; *p* = 0.173) [16, 17]. This dissociation is most plausibly explained by the high prevalence of non–disease-related mortality in meningioma populations. Data from the French national database reported a 5-year cumulative incidence of meningioma-related death of 2.85%, compared with 6.3% for competing non–disease-related mortality, with the latter being 2.21-fold more frequent [20, 21]. Consistent with these findings, 84.2% of deaths in the early RT group in our study were attributable to non–tumor-related causes, underscoring the necessity of competing risk methodology for the accurate interpretation of survival outcomes in grade 2 meningioma studies.

Recurrent meningiomas may arise from the dural margins of the initial resection, highlighting the importance of accurately defining surgical extent [22]. The Simpson grading system, introduced in 1957, classifies resection based on dural involvement and has been consistently associated with recurrence risk, with reported rates of 9%, 19%, 29%, and 44% following grades I–IV resections, respectively [10, 23]. Consistent with prior reports, Przybylowski et al. demonstrated superior recurrence-free survival with Simpson grade I resection compared with grades II–IV (*p* < 0.01) [24]. In our cohort, Simpson grade II resection was achieved more frequently in patients without recurrence than in those who recurred (39.0% vs 15.9%, *p* = 0.002), further supporting the link between surgical resection and disease control.

Despite its widespread use, the Simpson classification remains controversial in the modern neurosurgical era. Chotai and Schwartz have suggested that postoperative MRI–based assessment of residual tumor volume may more accurately reflect surgical extent [25]. Supporting this view, Gillespie et al. reported that 56% of residual tumors progressed over a median 64-month follow-up, with tumor grade, rather than residual volume, emerging as the dominant predictor of growth [26]. Moreover, because extent of resection is influenced by tumor location and size, the clinical reliability of the Simpson grading system continues to be debated [27]. Consistent with these observations, subtotal resection was performed in approximately three-quarters of sphenoid wing and parasellar tumors in our cohort (*p* < 0.001), underscoring the anatomic constraints that frequently limit complete resection in these regions.

Accumulating evidence indicates that tumor proliferative activity, as reflected by the Ki-67/MIB-1 index and mitotic count, represents a central biological determinant of outcome in grade 2 meningiomas. Prior studies have consistently demonstrated the prognostic significance of Ki-67, with Wang et al. identifying MIB-1 as an independent predictor of progression in a cohort of 263 atypical meningioma patients (HR 2.64, *p* < 0.001) [28], and Shukla et al. defining an optimal Ki-67 cutoff of 4.1%, above which patients experienced significantly higher risks of both recurrence (HR 2.9, *p* = 0.009) and mortality (HR 2.8, *p* = 0.036) [29]. Extending these observations, Ki-67 remained independently associated with long-term disease control in our cohort, emerging as a significant predictor of 10-year progression-free survival after multivariable adjustment (aHR 1.05, *p* = 0.018).

Complementing proliferative indices, mitotic activity has been shown to capture aggressive tumor behavior beyond surgical extent alone. Byun et al. reported that a mitotic count ≥ 6 per 10 high-power fields significantly increased progression risk even following gross total resection [30], while Kwon et al. demonstrated a strong association between high mitotic index and anaplastic transformation (OR 1.44, *p* = 0.004) [31]. In concordance with these findings, mitotic count independently predicted early disease control in our study, retaining prognostic significance for 3-year progression-free survival. Collectively, these data reinforce the concept that biological aggressiveness, as defined by proliferative and mitotic parameters, drives both early and late progression, underscoring the need to integrate tumor biology into postoperative risk stratification and treatment decision-making.

Brain invasion has been consistently recognized as a well-established risk factor for recurrence in grade 2 meningiomas [32–36] and was incorporated as a diagnostic criterion in the WHO 2016 classification, before being excluded again in the WHO 2021 update. In our cohort, brain invasion was significantly associated with 10-year progression-free survival on univariable analysis; however, this association did not remain significant after multivariable adjustment, suggesting that its prognostic impact may be mediated through, or confounded by, other tumor- and treatment-related factors.

Male sex has been identified as an adverse prognostic factor in meningioma across multiple studies. In a population-based analysis using the SEER database, Recker et al. reported male sex as an independent risk factor in patients with atypical meningioma [37]. Consistent with these findings, male sex emerged as a significant prognostic factor for OS in our cohort (aHR 2.69, *p* = 0.040). Advanced age has likewise been consistently associated with inferior outcomes in both our study and the existing literature. Rautalin et al. demonstrated that meningioma patients aged ≥ 80 years had a 2.5-fold higher mortality risk within the first postoperative year compared with the general population [38]. In line with these observations, age remained a significant predictor of OS in both univariable and multivariable analyses in our cohort.

With respect to RT fractionation, we observed that conventional fractionation was associated with superior PFS compared with hypofractionated schedules and SRS/fSRS. This finding is noteworthy when contrasted with the 47% 3-year progression-free survival reported in the fractionated radiosurgery series by Marchetti et al. [39]. However, this apparent advantage should be interpreted with caution, as patients treated with conventional fractionation in our study were predominantly drawn from the adjuvant RT group, raising the possibility that treatment timing, rather than fractionation alone, may have driven the observed difference in outcomes.

This study has several notable methodological strengths. First, the large sample size of 263 patients and the extended follow-up period (2005–2023) enabled robust and reliable survival estimates. Second, competing risks of death were explicitly accounted for using the Fine–Gray subdistribution hazard model, in accordance with the methodological standards proposed by Champeaux-Depond et al. [20, 21]. Third, to address missing data, MICE was applied to variables with substantial missingness—Ki-67 (40.7%) and mitotic count (39.9%)—thereby minimizing the loss of statistical power and potential bias associated with complete-case analyses. Still, given the use of multiple imputation to address missing data, the results should be interpreted in the context of imputation-based modeling. Fourth, time-dependent covariate analyses were performed to evaluate violations of the proportional hazards assumption. The observation that the hazard ratio associated with RT timing decreased over time (aHR: 12.56 → 3.31) suggests that early RT confers a pronounced initial benefit, which attenuates during longer follow-up.

Several important limitations should be acknowledged. First, the retrospective design precludes complete control of selection bias and residual confounding. In particular, it cannot be excluded that biologically more aggressive tumors were preferentially allocated to the late RT group. In addition, the results may be somewhat overestimated because patients who underwent surgery and never experienced recurrence were excluded from the analysis. Nevertheless, the median follow-up duration exceeded 7 years, and the majority of events occurred within the first 10 years of follow-up. Moreover, the observed PFS outcomes are consistent with historical controls. Therefore, despite these limitations, the findings remain clinically meaningful, although they should be interpreted with appropriate caution. Another important limitation is the high rate of missing pathological data, particularly for Ki-67 and mitotic count. Although multiple imputation was applied to address this issue, imputed values may not fully reflect the true biological measurements. Furthermore, TERT promoter mutation status was unavailable for 70.2% of patients, which precluded contemporary molecular risk stratification. Treatment-era heterogeneity spanning nearly two decades (2005–2023) represents an additional limitation, as substantial advances occurred during this period in radiotherapy techniques, surgical approaches, and pathological assessment standards. Changes in diagnostic criteria, particularly the updates introduced in the 2016 and 2021 WHO classifications, may also have influenced outcome interpretation. Finally, given the absence of randomization, it cannot be definitively determined whether the observed differences in progression-free survival are attributable solely to the timing of RT or are partly related to patient selection effects.

## Conclusion

In this large national multicenter cohort of patients with WHO grade 2 meningiomas, adjuvant RT was associated with a substantial and durable improvement in PFS compared with late RT, including in patients who underwent GTR. This benefit persisted across short-, intermediate-, and long-term follow-up, whereas no corresponding OS advantage was observed, likely reflecting the high burden of competing non–tumor-related mortality in this population. Tumor biology—particularly mitotic activity and proliferative indices—emerged as key determinants of both early and late disease control, underscoring the limitations of relying on surgical extent alone for postoperative risk stratification. Collectively, these findings support the consideration of postoperative RT as a disease-control strategy in selected patients with grade 2 meningiomas, even after GTR, and highlight the importance of integrating tumor biology, competing-risk methodology, and RT timing into individualized treatment decision-making. Prospective randomized trials are awaited to definitively refine patient selection and optimize treatment sequencing.

## Electronic supplementary material

Below is the link to the electronic supplementary material.

Supplementary material 1

Supplementary material 2

Supplementary material 3

Supplementary material 4

## Data Availability

Research data are stored in an institutional repository and will be shared upon request to the corresponding author.
