# Supplementary material for: Outcomes of early versus late radiotherapy in grade 2 meningiomas: a National retrospective analysis from the TROD neuro-oncology group
Source: J Neurooncol. 2026 May 5;177(3):133. doi: 10.1007/s11060-026-05590-8 (PMC13144216; doi:10.1007/s11060-026-05590-8)
Supplement: Supplementary file 1 — Supplementary material 1 [file 11060_2026_5590_MOESM1_ESM.pdf]

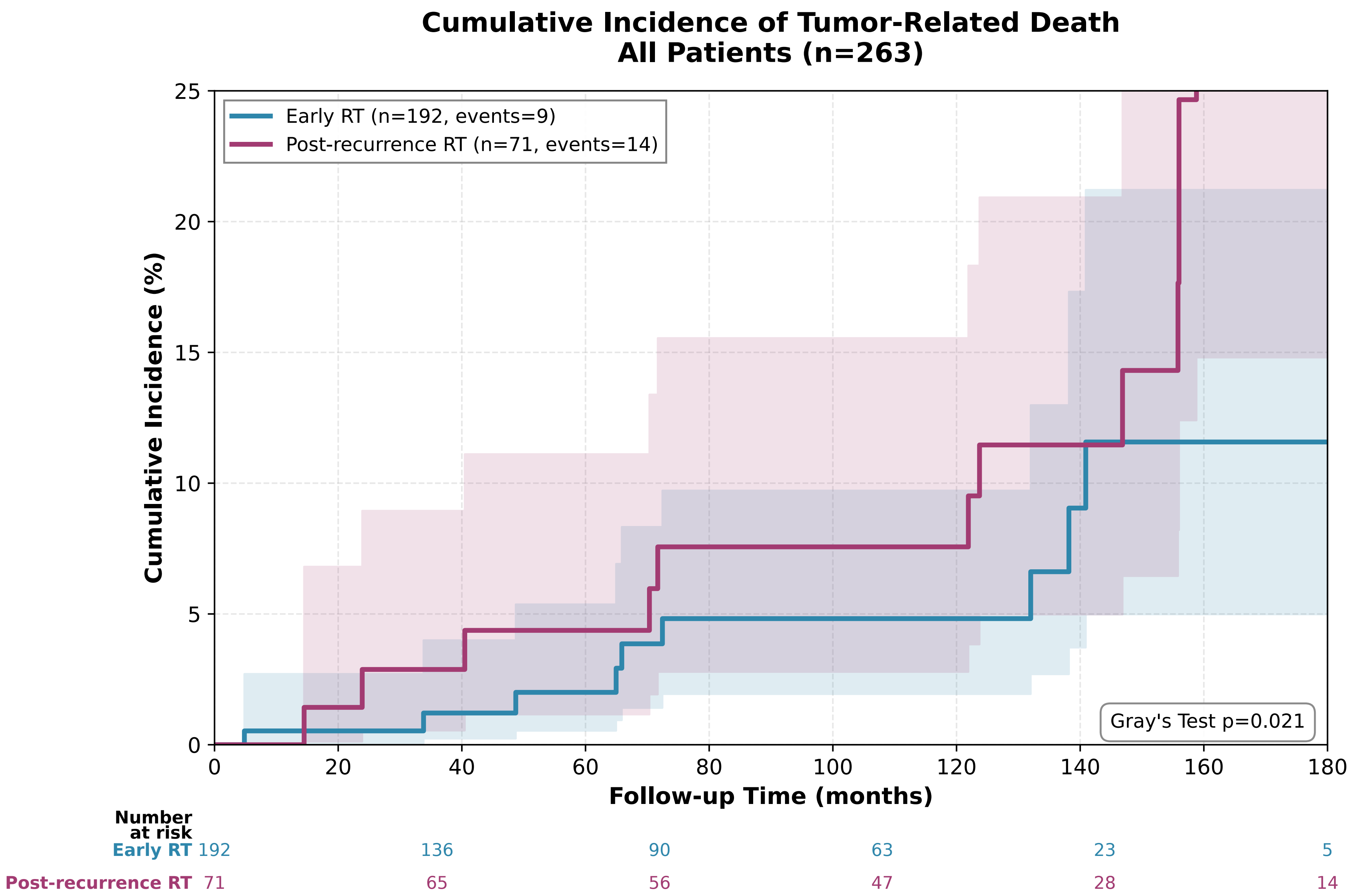
Cumulative Incidence of Tumor-Related Death
All Patients (n=263)
Early RT (n=192, events=9)
Post-recurrence RT (n=71, events=14)
Cumulative Incidence (%)
0
5
10
15
20
25
0
20
40
60
80
100
120
140
160
180
Follow-up Time (months)
Gray's Test p=0.021
Number at risk
Early RT 192 136 90 63 23 5
Post-recurrence RT 71 65 56 47 28 14
