# Supplementary material for: Outcomes of early versus late radiotherapy in grade 2 meningiomas: a National retrospective analysis from the TROD neuro-oncology group
Source: J Neurooncol. 2026 May 5;177(3):133. doi: 10.1007/s11060-026-05590-8 (PMC13144216; doi:10.1007/s11060-026-05590-8)
Supplement: Supplementary file 2 — Supplementary material 2 [file 11060_2026_5590_MOESM2_ESM.pdf]

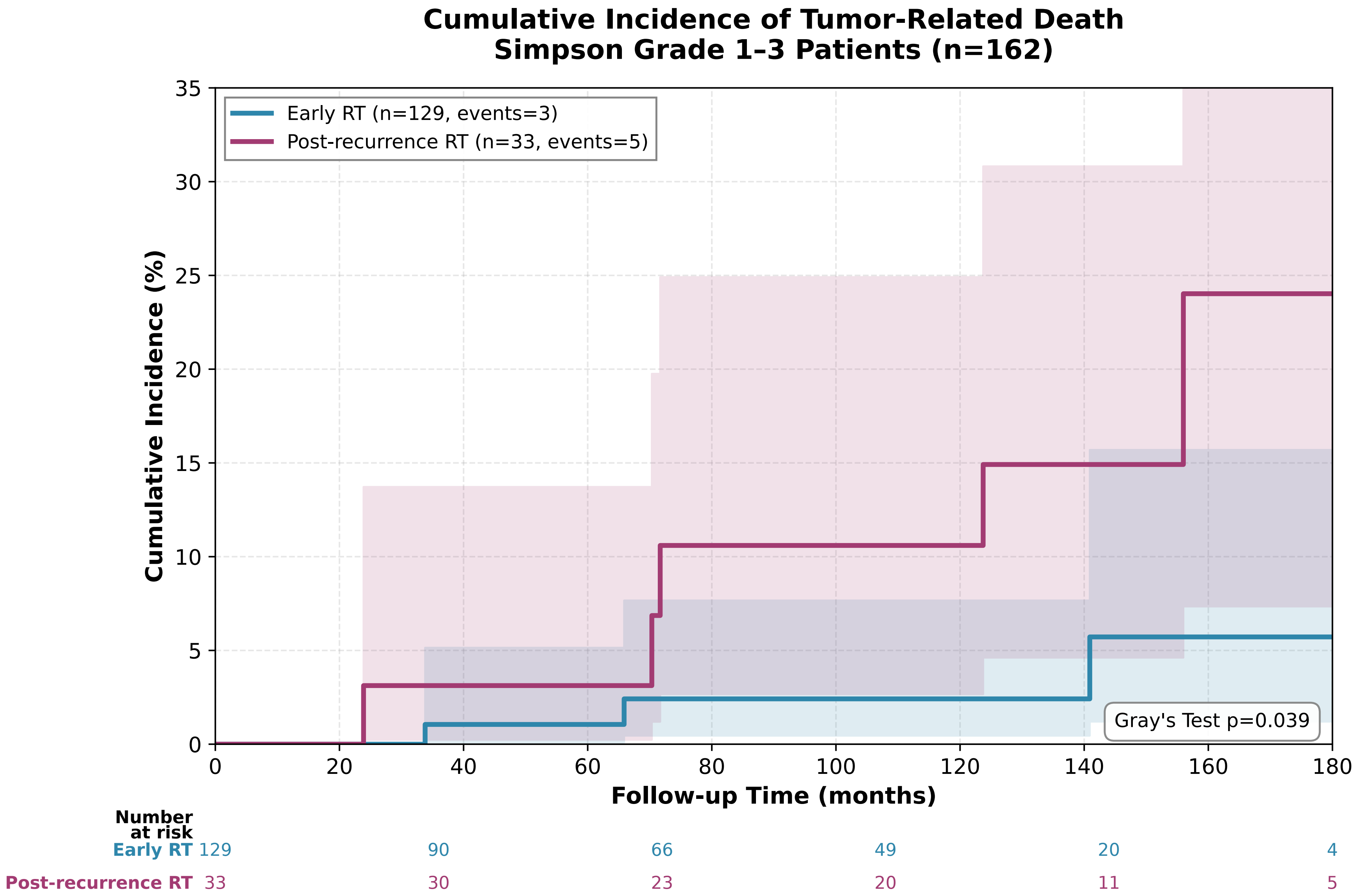
Cumulative Incidence of Tumor-Related Death
Simpson Grade 1–3 Patients (n=162)
Early RT (n=129, events=3)
Post-recurrence RT (n=33, events=5)
Gray's Test p=0.039
Cumulative Incidence (%)
0
5
10
15
20
25
30
35
Follow-up Time (months)
0
20
40
60
80
100
120
140
160
180
Number at risk
Early RT 129 90 66 49 20 4
Post-recurrence RT 33 30 23 20 11 5
