# Supplementary material for: Outcomes of early versus late radiotherapy in grade 2 meningiomas: a National retrospective analysis from the TROD neuro-oncology group
Source: J Neurooncol. 2026 May 5;177(3):133. doi: 10.1007/s11060-026-05590-8 (PMC13144216; doi:10.1007/s11060-026-05590-8)
Supplement: Supplementary file 3 — Supplementary material 3 [file 11060_2026_5590_MOESM3_ESM.docx]

**Table-1.** Comparison by Recurrence Status After Gross Total Resection (Simpson Grade 1–3)

| **Variables** |  | **Recurrent (n=118)** | **Non-recurrent (n=44)** | **p** |
| --- | --- | --- | --- | --- |
| **Age** *(year)* |  | 54,2 ± 12,1 | 52,6 ± 14,9 | 0,525 |
| **Age group** | ≤50 yaş | 48 (40,7) | 20 (45,5) | 0,712 |
|  | >50 yaş | 70 (59,3) | 24 (54,5) |  |
| **Gender** | Female | 69 (58,5) | 24 (54,5) | 0,786 |
|  | Male | 49 (41,5) | 20 (45,5) |  |
| **ECOG Performance Score** | 0 | 67 (58,3) | 18 (42,9) | 0,257 |
|  | 1 | 39 (33,9) | 20 (47,6) |  |
|  | 2 | 8 (7,0) | 3 (7,1) |  |
|  | 3 | 1 (0,9) | 1 (2,4) |  |
| **Tumor size** *(mm)* |  | 45,0 [16,0–90,0] | 40,0 [13,0–120,0] | 0,333 |
| **Tumor Location** | Convexity | 57 (48,7) | 11 (25,0) | 0,085 |
|  | Falx/Parasagittal | 29 (24,8) | 16 (36,4) |  |
|  | Sphenoidwing/Parasellar | 7 (6,0) | 6 (13,6) |  |
|  | Olfactory Sulcus | 9 (7,7) | 3 (6,8) |  |
|  | Posterior Fossa | 4 (3,4) | 2 (4,5) |  |
|  | Other | 11 (9,4) | 6 (13,6) |  |
| **Simpson grade** | Grade 1 | 53 (44,9) | 20 (45,5) | **0,002** |
|  | Grade 2 | 46 (39,0) | 7 (15,9) |  |
|  | Grade 3 | 19 (16,1) | 17 (38,6) |  |
| **Pathology** | Atypical | 113 (95,8) | 43 (97,7) | 0,999 |
|  | Chordoid | 5 (4,2) | 1 (2,3) |  |
| **Ki-67 Proliferation Index** *(%)* |  | 10,0 [1,0–75,0] | 12,0 [2,0–45,0] | 0,147 |
| **Mitosis** *(/10 HPF)* |  | 4,5 [2,0–57,0] | 8,0 [2,0–30,0] | **0,004** |
| **Brain Invasion,** *yes* |  | 78 (70,9) | 26 (65,0) | 0,621 |
| **TERT Mutation** | Yes | 28 (25,9) | 20 (50,0) | **0,002** |
|  | No | 1 (0,9) | 2 (5,0) |  |
|  | Unknown | 79 (73,1) | 18 (45,0) |  |
| **Anaplasia,** *yes* |  | 3 (2,9) | 0 (0,0) | 0,570 |
| **Hypercellularity,** *yes* |  | 50 (49,0) | 21 (60,0) | 0,355 |
| **Necrosis,** *yes* |  | 70 (68,0) | 24 (63,2) | 0,737 |
| **RT technique** | 3D-CRT | 24 (20,3) | 8 (18,2) | 0,932 |
|  | IMRT/VMAT | 94 (79,7) | 36 (81,8) |  |
| **Total Dose** *(Gy)* |  | 54,0 [12,0–60,0] | 51,0 [13,0–66,0] | **<0,001** |
| **Fraction number** |  | 30,0 [1,0–33,0] | 26,0 [1,0–33,0] | **<0,001** |
| **RT fractionation** | Conventional | 110 (93,2) | 22 (50,0) | **<0,001** |
|  | Hypofractionated | 3 (2,5) | 14 (31,8) |  |
|  | SRS/fSRS | 5 (4,2) | 8 (18,2) |  |
| **RT timing** | Early RT | 118 (100,0) | 11 (25,0) | **<0,001** |
|  | Late RT | 0 (0,0) | 33 (75,0) |  |
| **Steroid use,** *yes* |  | 81 (80,2) | 17 (53,1) | **0,005** |
| **Antiepileptic use,** *yes* |  | 55 (51,4) | 15 (42,9) | 0,495 |
| **Cause of death** | Non-tumoral | 16 (100,0) | 3 (27,3) | **<0,001** |
|  | Tumor | 0 (0,0) | 8 (72,7) |  |
| **Disease related death,** *yes* |  | 0 (0,0) | 8 (19,5) | **<0,001** |
| **Death From Any Cause,** *yes* |  | 16 (13,6) | 11 (25,0) | **0,099** |

**Bolded** p values indicate statistical significance (p ≤ 0.05). Continuous variables are presented as mean ± standard deviation for normally distributed data and median [minimum–maximum] for non–normally distributed data. Categorical variables are reported as n (%). PFS, progression-free survival; OS, overall survival; ECOG, Eastern Cooperative Oncology Group; HPF, high-power field; TERT, telomerase reverse transcriptase; 3D-CRT, three-dimensional conformal radiotherapy; IMRT, intensity-modulated radiotherapy; VMAT, volumetric modulated arc therapy; SRS, stereotactic radiosurgery; fSRS, fractionated stereotactic radiosurgery; RT, radiotherapy.

**Table-2.** Patient, Tumor, and Treatment Characteristics by Overall Survival Status

| **Variables** |  | **Alive (n=210)** | **Exitus (n=53)** | **p** |
| --- | --- | --- | --- | --- |
| **Age** *(year)* |  | 52,0 ± 13,1 | 60,8 ± 13,4 | **<0,001** |
| **Age group** | ≤50 | 96ᵃ (45,7) | 11ᵇ (20,8) | **0,001** |
|  | >50 | 114ᵃ (54,3) | 42ᵇ (79,2) |  |
| **Gender** | Female | 128ᵃ (61,0) | 24ᵇ (45,3) | **0,039** |
|  | Male | 82ᵃ (39,0) | 29ᵇ (54,7) |  |
| **ECOG Performance Score** | 0 | 107ᵃ (52,2) | 17ᵇ (35,4) | 0,090 |
|  | 1 | 80 (39,0) | 22 (45,8) |  |
|  | 2 | 15 (7,3) | 7 (14,6) |  |
|  | 3 | 3 (1,5) | 2 (4,2) |  |
| **Tumor size** *(mm)* |  | 44,9 ± 18,6 | 48,6 ± 12,4 | 0,239 |
| **Tumor Location** | Convexity | 72 (34,3) | 16 (31,4) | 0,566 |
|  | Falx/Parasagittal | 50 (23,8) | 12 (23,5) |  |
|  | Sphenoidwing/Parasellar | 39 (18,6) | 13 (25,5) |  |
|  | Olfactory Sulcus | 13 (6,2) | 2 (3,9) |  |
|  | Posterior Fossa | 9 (4,3) | 0 (0,0) |  |
|  | Other | 27 (12,9) | 8 (15,7) |  |
| **Extent of surgery** | Simpson 1–3 | 135 (64,3) | 27 (50,9) | 0,074 |
|  | Simpson 4–5 | 75 (35,7) | 26 (49,1) |  |
| **Simpson Grade** | Grade 1 | 65ᵃ (31,0) | 8ᵇ (15,1) | 0,114 |
|  | Grade 2 | 40 (19,0) | 13 (24,5) |  |
|  | Grade 3 | 30 (14,3) | 6 (11,3) |  |
|  | Grade 4 | 70 (33,3) | 23 (43,4) |  |
|  | Grade 5 | 5 (2,4) | 3 (5,7) |  |
| **Pathology** | Atypical | 200 (95,2) | 52 (98,1) | 0,350 |
|  | Chordoid | 10 (4,8) | 1 (1,9) |  |
| **Ki-67 Proliferation Index** *(%)* |  | 10,0 [1,0–75,0] | 14,0 [2,0–30,0] | 0,061 |
| **Mitosis** *(/10 BBA)* |  | 5,0 [0,0–57,0] | 4,0 [2,0–30,0] | 0,587 |
| **Brain Invasion,** *yes* |  | 75ᵃ (38,3) | 27ᵇ (57,4) | **0,017** |
| **TERT Mutation** | Yes | 58 (30,1) | 8 (19,0) | 0,344 |
|  | No | 3 (1,6) | 1 (2,4) |  |
|  | Unknown | 132 (68,4) | 33 (78,6) |  |
| **Anaplasia,** *yes* |  | 4 (2,3) | 2 (5,1) | 0,327 |
| **Hypercellularity,** *yes* |  | 78 (46,2) | 17 (48,6) | 0,794 |
| **Necrosis,** *yes* |  | 54 (30,9) | 13 (34,2) | 0,687 |
| **RT technique** | 3D-CRT | 35ᵃ (16,7) | 18ᵇ (34,0) | **0,005** |
|  | IMRT/VMAT | 175ᵃ (83,3) | 35ᵇ (66,0) |  |
| **Total Dose** *(Gy)* |  | 54,0 [12,0–66,0] | 54,0 [20,0–66,0] | 0,254 |
| **Fraction number** |  | 30,0 [1,0–33,0] | 30,0 [5,0–33,0] | 0,341 |
| **RT fractionation** | Conventional | 159 (75,7) | 39 (73,6) | 0,362 |
|  | Hypofractionated | 30 (14,3) | 11 (20,8) |  |
|  | SRS/fSRS | 21 (10,0) | 3 (5,7) |  |

**Bolded** p values indicate statistical significance (p ≤ 0.05). Continuous variables are presented as mean ± standard deviation for normally distributed data and median [minimum–maximum] for non–normally distributed data. Categorical variables are reported as n (%). Different superscript letters (ᵃ, ᵇ) within the same row indicate statistically significant differences between groups. PFS, progression-free survival; OS, overall survival; ECOG, Eastern Cooperative Oncology Group; HPF, high-power field; TERT, telomerase reverse transcriptase; 3D-CRT, three-dimensional conformal radiotherapy; IMRT, intensity-modulated radiotherapy; VMAT, volumetric modulated arc therapy; SRS, stereotactic radiosurgery; fSRS, fractionated stereotactic radiosurgery; RT, radiotherapy.

**Table 3.** Comparison by Overall Survival Status After Gross Total Resection (Simpson Grade 1–3)

| **Variables** |  | **Alive (n=135)** | **Exitus (n=27)** | **p** |
| --- | --- | --- | --- | --- |
| **Age** *(year)* |  | 52,4 ± 12,8 | 60,6 ± 11,5 | **0,002** |
| **Age group** | ≤50 | 61 (45,2) | 7 (25,9) | 0,064 |
|  | >50 | 74 (54,8) | 20 (74,1) |  |
| **Gender** | Female | 81 (60,0) | 12 (44,4) | 0,136 |
|  | Male | 54 (40,0) | 15 (55,6) |  |
| **ECOG Performance Score** | 0 | 77ᵃ (57,9) | 8ᵇ (33,3) | 0,089 |
|  | 1 | 47 (35,3) | 12 (50,0) |  |
|  | 2 | 8 (6,0) | 3 (12,5) |  |
|  | 3 | 1 (0,8) | 1 (4,2) |  |
| **Tumor size** *(mm)* |  | 44,0 ± 17,4 | 48,0 ± 12,4 | 0,308 |
| **Tumor Location** | Convexity | 57 (42,2) | 11 (42,3) | 0,723 |
|  | Falx/Parasagittal | 38 (28,1) | 7 (26,9) |  |
|  | Sphenoidwing/Parasellar | 10 (7,4) | 3 (11,5) |  |
|  | Olfactory Sulcus | 11 (8,1) | 1 (3,8) |  |
|  | Posterior Fossa | 6 (4,4) | 0 (0,0) |  |
|  | Other | 13 (9,6) | 4 (15,4) |  |
| **Simpson Grade** | Grade 1 | 65 (48,1) | 8 (29,6) | 0,131 |
|  | Grade 2 | 40 (29,6) | 13 (48,1) |  |
|  | Grade 3 | 30 (22,2) | 6 (22,2) |  |
| **Pathology** | Atypical | 129 (95,6) | 27 (100,0) | 0,264 |
|  | Chordoid | 6 (4,4) | 0 (0,0) |  |
| **Ki-67 Proliferation Index** *(%)* |  | 10,0 [1,0–75,0] | 15,5 [2,0–24,0] | 0,211 |
| **Mitosis** *(/10 BBA)* |  | 5,0 [2,0–57,0] | 7,0 [2,0–30,0] | 0,174 |
| **Brain Invasion,** *yes* |  | 37 (29,1) | 9 (39,1) | 0,339 |
| **TERT Mutation** | Yes | 42 (33,6) | 6 (26,1) | 0,569 |
|  | No | 2 (1,6) | 1 (4,3) |  |
|  | Unknown | 81 (64,8) | 16 (69,6) |  |
| **Anaplasia,** *yes* |  | 2 (1,7) | 1 (4,8) | 0,365 |
| **Hypercellularity,** *yes* |  | 55 (47,8) | 11 (50,0) | 0,852 |
| **Necrosis,** *yes* |  | 40 (33,6) | 7 (31,8) | 0,870 |
| **RT technique** | 3D-CRT | 20ᵃ (14,8) | 12ᵇ (44,4) | **<0,001** |
|  | IMRT/VMAT | 115ᵃ (85,2) | 15ᵇ (55,6) |  |
| **Total Dose** *(Gy)* |  | 54,0 [12,0–66,0] | 54,0 [20,0–60,0] | 0,182 |
| **Fraction number** |  | 30,0 [1,0–33,0] | 30,0 [5,0–30,0] | 0,310 |
| **RT fractionation** | Conventional | 111 (82,2) | 21 (77,8) | 0,247 |
|  | Hypofractionated | 12 (8,9) | 5 (18,5) |  |
|  | SRS/fSRS | 12 (8,9) | 1 (3,7) |  |

**Bolded** p values indicate statistical significance (p ≤ 0.05). Continuous variables are presented as mean ± standard deviation for normally distributed data and median [minimum–maximum] for non–normally distributed data. Categorical variables are reported as n (%). Different superscript letters (ᵃ, ᵇ) within the same row indicate statistically significant differences between groups. PFS, progression-free survival; OS, overall survival; ECOG, Eastern Cooperative Oncology Group; HPF, high-power field; TERT, telomerase reverse transcriptase; 3D-CRT, three-dimensional conformal radiotherapy; IMRT, intensity-modulated radiotherapy; VMAT, volumetric modulated arc therapy; SRS, stereotactic radiosurgery; fSRS, fractionated stereotactic radiosurgery; RT, radiotherapy.

**Table 4.** Prognostic Factors for All-Cause Mortality in the Overall Cohort: Cox Regression Analysis (n=263)

| **Variables** | **Univariate** | | **Multivariate** | |
| --- | --- | --- | --- | --- |
|  | **HR [%95 CI]** | **p** | **aHR [%95 CI]** | **p** |
| **Age** *(year)* | 1,07 [1,04–1,09] | **<0,001** | 1,07 [1,04–1,09] | **<0,001** |
| **Gender** |  |  |  |  |
| Female *(ref)* | 1 | — | 1 | — |
| Male | 2,16 [1,25–3,72] | **0,006** | 1,83 [1,05–3,21] | **0,035** |
| **Extent of surgical resection** |  |  |  |  |
| Simpson grade 1–3 *(ref)* | 1 | — | 1 | — |
| Simpson grade 4–5 | 1,44 [0,84–2,48] | 0,184 | 1,88 [1,06–3,36] | **0,032** |
| **RT timing** |  |  |  |  |
| Early RT *(ref)* | 1 | — | 1 | — |
| Late RT | 0,73 [0,41–1,30] | 0,285 | 0,67 [0,36–1,27] | 0,221 |
| **Tumor location** |  |  |  |  |
| Supratentorial Non-Base *(ref)* | 1 | — | — | — |
| Supratentorial Skull Base | 0,90 [0,49–1,64] | 0,72 | — | — |
| Posterior Fossa/Other | 0,67 [0,32–1,42] | 0,296 | — | — |
| **Ki-67 Proliferation Index** *(%)* | 1,04 [1,00–1,07] | 0,05 | 1,03 [0,99–1,08] | 0,193 |
| **Mitosis** *(/10 BBA)* | 1,00 [0,95–1,06] | 0,991 | — | — |
| **Necrosis,** *yes* | 1,23 [0,67–2,28] | 0,503 | — | — |
| **Brain Invasion,** *yes* | 1,58 [0,92–2,73] | 0,099 | — | — |
| **Total Dose** *(Gy)* | 1,01 [0,99–1,03] | 0,372 | — | — |
| **RT Fractionation** |  |  |  |  |
| Conventional *(ref)* | 1 | — | — | — |
| Hypofractionated | 0,67 [0,34–1,32] | 0,248 | — | — |
| SRS/fSRS | 0,64 [0,20–2,05] | 0,448 | — | — |
| **RT Technique** |  |  |  |  |
| 3D-CRT *(ref)* | 1 | — | 1 | — |
| IMRT/VMAT | 1,19 [0,66–2,14] | 0,556 | 1,20 [0,65–2,22] | 0,553 |

**Model statistics:** number of events = 53; **C-index = 0.737**; **likelihood ratio test:** χ² = 44.83, *p* < 0.001; **global Schoenfeld test:** χ² = 6.00, df = 6, *p* = 0.423. **Bolded p values indicate statistical significance (p ≤ 0.05).** Variables with *p* < 0.10 in univariable analyses and clinically relevant variables (treatment group and radiotherapy technique) were included in the multivariable model. **HR**, hazard ratio; **aHR**, adjusted hazard ratio; **CI**, confidence interval; **RT**, radiotherapy; **HPF**, high-power field; **3D-CRT**, three-dimensional conformal radiotherapy; **IMRT**, intensity-modulated radiotherapy; **VMAT**, volumetric modulated arc therapy; **SRS**, stereotactic radiosurgery; **fSRS**, fractionated stereotactic radiosurgery.

**Table 5.** Cause-Specific Cox Regression Analysis for Tumor-Related Death in the Overall Cohort (n=263)

| **Variables** | **Univariate** | | **Multivariate** | |
| --- | --- | --- | --- | --- |
|  | **csHR [%95 CI]** | **p** | **acsHR [%95 CI]** | **p** |
| **Age** *(year)* | 1,03 [1,00–1,07] | 0,085 | 1,04 [1,00–1,07] | **0,049** |
| **Gender** |  |  |  |  |
| Female *(ref)* | 1 | — | 1 | — |
| Male | 3,01 [1,29–7,01] | **0,011** | 2,90 [1,22–6,91] | **0,016** |
| **Extent of surgical resection** |  |  |  |  |
| Simpson grade 1–3 *(ref)* | 1 | — | 1 | — |
| Simpson grade 4–5 | 2,65 [1,12–6,29] | **0,026** | 2,49 [0,96–6,43] | 0,06 |
| **RT timing** |  |  |  |  |
| Early RT *(ref)* | 1 | — | 1 | — |
| Late RT | 1,86 [0,78–4,45] | 0,162 | 1,55 [0,60–4,00] | 0,37 |
| **Tumor location** |  |  |  |  |
| Supratentorial Non-Base *(ref)* | 1 | — | — | — |
| Supratentorial Skull Base | 1,26 [0,53–2,96] | 0,601 | — | — |
| Posterior Fossa/Other | 1,03 [0,38–2,78] | 0,957 | — | — |
| **Ki-67 Proliferation Index** *(%)* | 1,04 [0,99–1,10] | 0,103 | — | — |
| **Mitosis** *(/10 BBA)* | 1,05 [1,00–1,10] | **0,042** | 1,06 [1,01–1,11] | **0,02** |
| **Necrosis,** *yes* | 1,68 [0,68–4,16] | 0,259 | — | — |
| **Brain Invasion,** *yes* | 2,17 [0,92–5,15] | 0,077 | — | — |
| **Total Dose** *(Gy)* | 0,98 [0,95–1,01] | 0,256 | — | — |
| **RT Fractionation** |  |  |  |  |
| Conventional *(ref)* | 1 | — | — | — |
| Hypofractionated | 1,23 [0,51–2,99] | 0,642 | — | — |
| SRS/fSRS | 1,14 [0,27–4,90] | 0,86 | — | — |
| **RT Technique** |  |  |  |  |
| 3D-CRT *(ref)* | 1 | — | — | — |
| IMRT/VMAT | 1,09 [0,45–2,60] | 0,852 | — | — |

**Model statistics:** tumor-related deaths = 23; **competing events (non–tumor-related deaths) = 30**; **C-index = 0.738**; **global Schoenfeld test:** *p* = 0.955. **Bolded p values indicate statistical significance (p ≤ 0.05).** In the cause-specific hazard model, non–tumor-related deaths were treated as censored events. This model is appropriate for **etiologic interpretation**. **csHR**, cause-specific hazard ratio; **acsHR**, adjusted cause-specific hazard ratio; **CI**, confidence interval; **RT**, radiotherapy; **HPF**, high-power field; **3D-CRT**, three-dimensional conformal radiotherapy; **IMRT**, intensity-modulated radiotherapy; **VMAT**, volumetric modulated arc therapy; **SRS**, stereotactic radiosurgery; **fSRS**, fractionated stereotactic radiosurgery.

**Table 6.** Fine–Gray Competing-Risk Regression Analysis for Tumor-Related Death in the Overall Cohort (n=263)

| **Variables** | **Univariate** | | **Multivariate** | |
| --- | --- | --- | --- | --- |
|  | **sHR [%95 CI]** | **p** | **asHR [%95 CI]** | **p** |
| **Age** *(year)* | 1,00 [0,97–1,03] | 0,873 | 1,01 [0,98–1,04] | 0,634 |
| **Gender** |  |  |  |  |
| Female *(ref)* | 1 | — | 1 | — |
| Male | 2,42 [1,05–5,59] | **0,039** | 2,70 [1,14–6,37] | **0,024** |
| **Extent of surgical resection** |  |  |  |  |
| Simpson grade 1–3 *(ref)* | 1 | — | 1 | — |
| Simpson grade 4–5 | 2,76 [1,17–6,52] | **0,02** | 2,55 [1,03–6,36] | **0,044** |
| **RT timing** |  |  |  |  |
| Early RT *(ref)* | 1 | — | 1 | — |
| Late RT | 2,70 [1,16–6,26] | **0,021** | 2,43 [0,99–5,97] | 0,052 |
| **Tumor location** |  |  |  |  |
| Supratentorial Non-Base *(ref)* | 1 | — | — | — |
| Supratentorial Skull Base | 1,59 [0,69–3,69] | 0,278 | — | — |
| Posterior Fossa/Other | 1,23 [0,46–3,32] | 0,682 | — | — |
| **Ki-67 Proliferation Index** *(%)* | 1,03 [0,98–1,09] | 0,252 | — | — |
| **Mitosis** *(/10 BBA)* | 1,05 [1,01–1,10] | **0,016** | 1,06 [1,01–1,11] | **0,019** |
| **Necrosis,** *yes* | 1,53 [0,63–3,74] | 0,346 | — | — |
| **Brain Invasion,** *yes* | 2,19 [0,93–5,17] | 0,074 | — | — |
| **Total Dose** *(Gy)* | 0,97 [0,94–1,00] | 0,073 | — | — |
| **RT Fractionation** |  |  |  |  |
| Conventional *(ref)* | 1 | — | — | — |
| Hypofractionated | 1,76 [0,74–4,17] | 0,198 | — | — |
| SRS/fSRS | 1,20 [0,28–5,16] | 0,803 | — | — |
| **RT Technique** |  |  |  |  |
| 3D-CRT *(ref)* | 1 | — | — | — |
| IMRT/VMAT | 0,82 [0,35–1,92] | 0,649 | — | — |

**Model statistics:** tumor-related deaths = 23; **competing events (non–tumor-related deaths) = 30**; **C-index = 0.747**.
**Bolded p values indicate statistical significance (p ≤ 0.05).** In the **Fine–Gray subdistribution hazard model**, patients experiencing competing events remain in the risk set. This model is appropriate for **prognostic assessment and estimation of cumulative incidence**. **sHR**, subdistribution hazard ratio; **asHR**, adjusted subdistribution hazard ratio; **CI**, confidence interval; **RT**, radiotherapy; **HPF**, high-power field; **3D-CRT**, three-dimensional conformal radiotherapy; **IMRT**, intensity-modulated radiotherapy; **VMAT**, volumetric modulated arc therapy; **SRS**, stereotactic radiosurgery; **fSRS**, fractionated stereotactic radiosurgery.

**Table 7.** Cumulative Incidence of Tumor-Related Death by RT Timing in the Overall Cohort (Aalen–Johansen Method)

| **RT timing** | **n** | **Event** | **Competing event** | **1-Year CIF *% (95% CI)*** | **3-Year CIF *% (95% CI)*** | **5-Year CIF *% (95% CI)*** | **10-Year CIF *% (95% CI)*** | **p** |
| --- | --- | --- | --- | --- | --- | --- | --- | --- |
| Early RT | 192 | 9 | 25 | 0,5 (0,0–2,7) | 1,2 (0,2–4,0) | 2,0 (0,5–5,4) | 4,8 (1,9–9,7) | **0,021** |
| Late RT | 71 | 14 | 5 | 1,4 (0,1–6,8) | 2,9 (0,5–8,9) | 4,4 (1,2–11,1) | 7,6 (2,8–15,5) |  |

**Bolded** p values indicate statistical significance (p ≤ 0.05). Cumulative incidence rates were estimated using the Aalen–Johansen method, with 95% confidence intervals shown in parentheses. Death from non–tumor-related causes was defined as the competing event. Between-group comparisons were performed using *p* values derived from the Fine–Gray subdistribution hazard model. CIF, cumulative incidence function; CI, confidence interval; RT, radiotherapy**.**

**Table 8.** Prognostic Factors for All-Cause Mortality After Gross Total Resection (Simpson Grade 1–3): Cox Regression Analysis (n=162)

| **Variables** | **Univariate** | | **Multivariate** | |
| --- | --- | --- | --- | --- |
|  | **HR [%95 CI]** | **p** | **aHR [%95 CI]** | **p** |
| **Age** *(year)* | 1,06 [1,02–1,09] | **0,001** | 1,06 [1,02–1,09] | **<0,001** |
| **Gender** |  |  |  |  |
| Female *(ref)* | 1 | — | 1 | — |
| Male | 2,18 [1,01–4,70] | **0,046** | 2,37 [1,07–5,24] | **0,033** |
| **RT timing** |  |  |  |  |
| Early RT *(ref)* | 1 | — | 1 | — |
| Late RT | 1,00 [0,44–2,29] | 0,996 | 1,18 [0,49–2,83] | 0,717 |
| **Tumor location** |  |  |  |  |
| Supratentorial Non-Base *(ref)* | 1 | — | — | — |
| Supratentorial Skull Base | 1,11 [0,38–3,23] | 0,841 | — | — |
| Posterior Fossa/Other | 0,66 [0,23–1,92] | 0,443 | — | — |
| **Ki-67 Proliferation Index** *(%)* | 1,03 [0,98–1,08] | 0,291 | — | — |
| **Mitosis** *(/10 BBA)* | 1,02 [0,96–1,08] | 0,579 | — | — |
| **Necrosis,** *yes* | 0,85 [0,36–2,01] | 0,707 | — | — |
| **Brain Invasion,** *yes* | 1,80 [0,82–3,94] | 0,144 | — | — |
| **Total Dose** *(Gy)* | 1,00 [0,97–1,04] | 0,916 | — | — |
| **RT Fractionation** |  |  |  |  |
| Conventional *(ref)* | 1 | — | — | — |
| Hypofractionated | 1,10 [0,42–2,93] | 0,844 | — | — |
| SRS/fSRS | 0,39 [0,05–2,91] | 0,362 | — | — |
| **RT Technique** |  |  |  |  |
| 3D-CRT *(ref)* | 1 | — | 1 | — |
| IMRT/VMAT | 0,72 [0,33–1,58] | 0,417 | 0,68 [0,30–1,51] | 0,342 |

**Model statistics:** number of events = 27; **EPV = 6.8**; **C-index = 0.656**; **likelihood ratio test:** χ² = 16.65, *p* = 0.002; **global Schoenfeld test:** χ² = 9.19, df = 4, *p* = 0.057. **Bolded p values indicate statistical significance (p ≤ 0.05).** A violation of the proportional hazards assumption was identified for **age** (Schoenfeld *p* = 0.007), indicating that the effect of age on mortality varies over time. **Treatment group** and **radiotherapy technique** were included in the multivariable model due to clinical relevance. **HR**, hazard ratio; **aHR**, adjusted hazard ratio; **CI**, confidence interval; **RT**, radiotherapy; **HPF**, high-power field; **EPV**, events per variable; **3D-CRT**, three-dimensional conformal radiotherapy; **IMRT**, intensity-modulated radiotherapy; **VMAT**, volumetric modulated arc therapy; **SRS**, stereotactic radiosurgery; **fSRS**, fractionated stereotactic radiosurgery

**Table 9.** Cause-Specific Hazard Regression Analysis for Tumor-Related Death in Patients Undergoing Gross Total Resection (Simpson Grade 1–3), With Non–Tumor-Related Deaths Censored (n=143)

| **Variables** | **Univariate** | | **Multivariate** | |
| --- | --- | --- | --- | --- |
|  | **csHR [%95 CI]** | **p** | **acsHR [%95 CI]** | **p** |
| **Age** *(year)* | 1,02 [0,96–1,08] | 0,557 | — | — |
| **Gender** |  |  |  |  |
| Female *(ref)* | 1 | — | — | — |
| Male | 3,33 [0,78–14,29] | 0,105 | — | — |
| **RT timing** |  |  |  |  |
| Early RT *(ref)* | 1 | — | 1 | — |
| Late RT | 3,71 [0,88–15,68] | 0,074 | 1,63 [0,62–4,28] | 0,317 |
| **Tumor location** |  |  |  |  |
| Supratentorial Non-Base *(ref)* | 1 | — | — | — |
| Supratentorial Skull Base | 0,85 [0,10–6,91] | 0,877 | — | — |
| Posterior Fossa/Other | 1,15 [0,23–5,87] | 0,867 | — | — |
| **Ki-67 Proliferation Index** *(%)* | 1,03 [0,94–1,12] | 0,529 | — | — |
| **Mitosis** *(/10 BBA)* | 1,05 [1,00–1,11] | **0,045** | 1,03 [0,97–1,09] | 0,309 |
| **Necrosis,** *yes* | 1,59 [0,37–6,75] | 0,529 | — | — |
| **Brain Invasion,** *yes* | 2,09 [0,48–8,97] | 0,323 | — | — |
| **Total Dose** *(Gy)* | 0,96 [0,92–1,01] | 0,105 | 0,99 [0,95–1,03] | 0,565 |
| **RT Fractionation** |  |  |  |  |
| Conventional *(ref)* | 1 | — | — | — |
| Hypofractionated | 2,83 [0,67–11,98] | 0,159 | — | — |
| SRS/fSRS | 1,31 [0,16–10,66] | 0,802 | — | — |
| **RT Technique** |  |  |  |  |
| 3D-CRT *(ref)* | 1 | — | — | — |
| IMRT/VMAT | 0,49 [0,12–2,08] | 0,333 | — | — |

**Model statistics:** tumor-related deaths = 8; **competing events (non–tumor-related deaths) = 19**; **EPV = 2.7**; **C-index = 0.791**; **likelihood ratio test:** χ² = 3.03, *p* = 0.387; **global Schoenfeld test:** χ² = 1.27, df = 3, *p* = 0.736. **Bolded p values indicate statistical significance (p ≤ 0.05).** In the cause-specific hazard model, non–tumor-related deaths were treated as censored events. Owing to the **low number of events** (n = 8) and **insufficient events per variable** (EPV = 2.7 < 5), results of the multivariable model should be considered **exploratory**, and the wide confidence intervals reflect limited statistical power. **csHR**, cause-specific hazard ratio; **acsHR**, adjusted cause-specific hazard ratio; **CI**, confidence interval; **RT**, radiotherapy; **HPF**, high-power field; **EPV**, events per variable; **3D-CRT**, three-dimensional conformal radiotherapy; **IMRT**, intensity-modulated radiotherapy; **VMAT**, volumetric modulated arc therapy; **SRS**, stereotactic radiosurgery; **fSRS**, fractionated stereotactic radiosurgery.

**Table 10.** Fine–Gray Subdistribution Hazard Regression Analysis for Tumor-Related Death in Patients Undergoing Gross Total Resection (Simpson Grade 1–3), Accounting for Competing Risks (n=162)

| **Variables** | **Univariate** | | **Multivariate** | |
| --- | --- | --- | --- | --- |
|  | **sHR [%95 CI]** | **p** | **asHR [%95 CI]** | **p** |
| **Age** *(year)* | 1,00 [0,94–1,05] | 0,889 | — | — |
| **Gender** |  |  |  |  |
| Female *(ref)* | 1 | — | — | — |
| Male | 2,57 [0,61–10,78] | 0,197 | — | — |
| **RT timing** |  |  |  |  |
| Early RT *(ref)* | 1 | — | 1 | — |
| Late RT | 4,52 [1,08–18,94] | **0,039** | 1,69 [0,66–4,31] | 0,273 |
| **Tumor location** |  |  |  |  |
| Supratentorial Non-Base *(ref)* | 1 | — | — | — |
| Supratentorial Skull Base | 0,83 [0,10–6,74] | 0,86 | — | — |
| Posterior Fossa/Other | 1,44 [0,29–7,21] | 0,655 | — | — |
| **Ki-67 Proliferation Index** *(%)* | 1,02 [0,94–1,12] | 0,598 | — | — |
| **Mitosis** *(/10 BBA)* | 1,06 [1,01–1,11] | **0,023** | 1,03 [0,98–1,09] | 0,272 |
| **Necrosis,** *yes* | 1,68 [0,40–7,08] | 0,479 | — | — |
| **Brain Invasion,** *yes* | 1,64 [0,39–6,88] | 0,501 | — | — |
| **Total Dose** *(Gy)* | 0,95 [0,91–1,00] | **0,048** | 0,99 [0,95–1,02] | 0,479 |
| **RT Fractionation** |  |  |  |  |
| Conventional *(ref)* | 1 | — | — | — |
| Hypofractionated | 3,28 [0,78–13,77] | 0,105 | — | — |
| SRS/fSRS | 1,77 [0,22–14,51] | 0,593 | — | — |
| **RT Technique** |  |  |  |  |
| 3D-CRT *(ref)* | 1 | — | — | — |
| IMRT/VMAT | 0,52 [0,13–2,13] | 0,361 | — | — |

**Model statistics:** tumor-related deaths = 8; **competing events (non–tumor-related deaths) = 19**; **EPV = 2.7**; **C-index = 0.812**; **likelihood ratio test:** χ² = 3.76, *p* = 0.288. **Bolded p values indicate statistical significance (p ≤ 0.05).** In the **Fine–Gray subdistribution hazard model**, patients experiencing competing events remained in the risk set. Owing to the **low number of events** (n = 8) and **insufficient events per variable** (EPV = 2.7 < 5), results of the multivariable model should be considered **exploratory**. Variables that were significant in univariable analyses (**treatment group, mitotic count, and total dose**) did not retain statistical significance in the multivariable model because of limited statistical power. **sHR**, subdistribution hazard ratio; **asHR**, adjusted subdistribution hazard ratio; **CI**, confidence interval; **RT**, radiotherapy; **HPF**, high-power field; **EPV**, events per variable; **3D-CRT**, three-dimensional conformal radiotherapy; **IMRT**, intensity-modulated radiotherapy; **VMAT**, volumetric modulated arc therapy; **SRS**, stereotactic radiosurgery; **fSRS**, fractionated stereotactic radiosurgery.

**Table 11.** Cumulative Incidence of Tumor-Related Death by RT Timing After Gross Total Resection (Aalen–Johansen Method)

| **RT timing** | **n** | **Event** | **Competing**  **event** | **1-Year CIF *% (95% CI)*** | **3-Year CIF *% (95% CI)*** | **5-Year CIF *% (95% CI)*** | **10-Year CIF *% (95% CI)*** | **p** |
| --- | --- | --- | --- | --- | --- | --- | --- | --- |
| Early RT | 129 | 3 | 16 | 0,0 (–) | 1,1 (0,1–5,1) | 1,1 (0,1–5,1) | 2,4 (0,5–7,7) | **0,039** |
| Late RT | 33 | 5 | 3 | 0,0 (–) | 3,1 (0,2–13,7) | 3,1 (0,2–13,7) | 10,6 (2,7–24,9) |  |

**Bolded** p values indicate statistical significance (p ≤ 0.05). Cumulative incidence rates were estimated using the Aalen–Johansen method, with 95% confidence intervals shown in parentheses. Death from non–tumor-related causes was defined as the competing event. Between-group comparisons were performed using *p* values derived from the Fine–Gray subdistribution hazard model. For 1-year CIF estimates, confidence intervals could not be calculated because of an insufficient number of events and are therefore indicated as not estimable (–). CIF, cumulative incidence function; CI, confidence interval; RT, radiotherapy**.**
