# Supplementary material for: Outcomes of early versus late radiotherapy in grade 2 meningiomas: a National retrospective analysis from the TROD neuro-oncology group
Source: J Neurooncol. 2026 May 5;177(3):133. doi: 10.1007/s11060-026-05590-8 (PMC13144216; doi:10.1007/s11060-026-05590-8)
Supplement: Supplementary file 4 — Supplementary material 4 [file 11060_2026_5590_MOESM4_ESM.docx]

**Supplemantary Figure 1.** Cumulative incidence curves for tumor-related mortality according to radiotherapy timing in the overall cohort (n = 263). Cumulative incidence estimates were calculated using the Aalen–Johansen method, with death from non–tumor-related causes treated as a competing event. Shaded areas represent 95% confidence intervals. The 10-year cumulative incidence of tumor-related mortality was 4.8% in the early radiotherapy group and 7.6% in the postrecurrence radiotherapy group (p = 0.021).

**Supplemantary Figure 2.** Cumulative incidence curves for tumor-related mortality according to radiotherapy timing in patients with Simpson grade 1–3 resection (n = 162). Cumulative incidence estimates were calculated using the Aalen–Johansen method, with death from non–tumor-related causes treated as a competing event. Shaded areas represent 95% confidence intervals. The 10-year cumulative incidence of tumor-related mortality was 2.4% in the early radiotherapy group and 10.6% in the postrecurrence radiotherapy group (p = 0.039).
